# Exceptional endocrine profiles characterise the meerkat: sex, status, and reproductive patterns

**DOI:** 10.1038/srep35492

**Published:** 2016-10-18

**Authors:** Charli S. Davies, Kendra N. Smyth, Lydia K. Greene, Debbie A. Walsh, Jessica Mitchell, Tim Clutton-Brock, Christine M. Drea

**Affiliations:** 1 Kalahari Research Trust, Kuruman River Reserve, Northern Cape, South Africa; 2 Department of Evolutionary Anthropology, Duke University, Durham, NC 27708, USA; 3 University Program in Ecology, Duke University, Durham, NC 27708, USA; 4 Mammal Research Institute, University of Pretoria, Pretoria, South Africa; 5 Department of Zoology, University of Cambridge, Cambridge, UK; 6 Department of Biology, Duke University, Durham, USA

## Abstract

In vertebrates, reproductive endocrine concentrations are strongly differentiated by sex, with androgen biases typifying males and estrogen biases typifying females. These sex differences can be reduced in female-dominant species; however, even the most masculinised of females have less testosterone (T) than do conspecific males. To test if aggressively dominant, female meerkats (*Suricata suricatta*) may be hormonally masculinised, we measured serum androstenedione (A_4_), T and estradiol (E_2_) in both sexes and social classes, during both ‘baseline’ and reproductive events. Relative to resident males, dominant females had greater A_4_, equivalent T and greater E_2_ concentrations. Males, whose endocrine values did not vary by social status, experienced increased T during reproductive forays, linking T to sexual behaviour, but not social status. Moreover, substantial E_2_ concentrations in male meerkats may facilitate their role as helpers. In females, dominance status and pregnancy magnified the unusual concentrations of measured sex steroids. Lastly, faecal androgen metabolites replicated the findings derived from serum, highlighting the female bias in total androgens. Female meerkats are thus strongly hormonally masculinised, possibly via A_4_’s bioavailability for conversion to T. These raised androgen concentrations may explain female aggressiveness in this species and give dominant breeders a heritable mechanism for their daughters’ competitive edge.

Sexual selection research has been heavily biased toward understanding Darwin’s mechanisms of intrasexual male competition and female choice; however, there has been growing appreciation for the potential of male choice, as well as mounting evidence of intense intrasexual competition in females^1^,^2^,^3^. Indeed, in some species, traditional sex roles, reflecting the prototypical dichotomy between male aggressiveness and female nurturance, are even reversed^4^,^5^. High energetic demands involved in gestation and lactation mean that competition for resources, such as access to food, shelter and care for offspring are particularly important in females^6^. In certain cooperatively breeding species that experience extreme resource competition, heightened female aggression in dominant breeders comes at a reproductive cost to subordinate helpers, resulting in greater reproductive skew between females than between males^1^,^7^,^8^. Nevertheless, the proximate mechanisms explaining these reversed sex differences remain poorly understood.

Sexual differentiation in mammalian development is underwritten, in large part, by sex differences in reproductive hormones, experienced pre- or peri-natally and throughout adulthood^9^,^10^. Quantitative differences in sex steroids are, therefore, ubiquitous, with a relative abundance of androgens, particularly testosterone (T: 17β-hydroxyandrost-4-en-3-one), typifying males and a relative abundance of estrogens, particularly estradiol (E_2_: 17β-estra-1,3,5(10)-triene-3,17-diol), typifying females^11^. In certain exceptional species characterised by heightened female aggression and/or by female social dominance over males, the traditional, adult endocrine sex difference in T generally maintains^12^,^13^,^14^,^15^,^16^. Therefore, explaining unusual female aggression or social dominance might require, among various possibilities, invoking unusual female exposure to prenatal hormones^17^ or enzyme activity^18^,^19^, altered adult receptor sensitivity^20^, or even the action of nontraditional steroids (potentially in either sex)^12^,^13^,^17^,^21^. Here, we seek to better characterise the hormonal substrates potentially associated with exceptional aggressiveness in the adult, dominant female meerkat (*Suricata suricatta*) by providing a more comprehensive examination of ‘heterologous’ hormones (i.e., various androgens in females and estrogens in males) in animals of both sexes, of both social classes, and in different reproductive phases.

According to the theory of mammalian sexual differentiation^9^,^10^, certain morphological and behavioural traits in females implicate hormonal masculinisation. In the best-known case of exceptional female aggressiveness^22^ and social dominance^23^, displayed by the spotted hyaena (*Crocuta crocuta*), female behavioural traits are associated with hormonal masculinisation^19^. Notably, year round concentrations of androstenedione (A_4_: androst-4-ene-3,17-dione), the androgenic precursor to T (as well as to estrogens), are greater in females than in males, but see ref. 24, and, during gestation, maternal A_4_ is readily converted to T by placental enzyme activity (involving 17ß-hydroxysteroid dehydrogenase)^17^,^18^,^19^. In spotted hyaenas, the accumulation of maternal T across gestation provides a basis for organisational effects on offspring behaviour^25^ that later, in adulthood, link to activational effects of hormones on aggression^26^. Because a hormonal mechanism of female masculinisation may provide a framework for understanding other female-dominant mammals (e.g. various strepsirrhine primates^16^,^27^,^28^), here we investigate whether or not there is hormonal potential for a similar mechanism to operate in the cooperatively breeding meerkat. Beyond appropriately timed T concentrations available prenatally (i.e., during gestation), one might expect evidence of T or its precursors circulating in adult females. Likewise, because a female-dominant social system could implicate male deference as a contributing mediating mechanism^29^,^30^, male endocrine profiles may also shed light on unusual sex-role reversals.

The meerkat is an obligate, cooperatively breeding carnivoran that lives in groups, called ‘clans,’ consisting of 3–50 individuals. Extreme reproductive skew is evident in both sexes^7^, but is particularly pronounced in females: although subordinates are physically able to breed and routinely become pregnant, dominant females produce over 80% of the pups surviving to independency^31^,^32^. Reproductive ‘suppression’ of subordinate females occurs predominantly through behavioural mediation, with dominant females impeding subordinates from producing surviving pups primarily through eviction, infanticide and resource competition^33^. Additionally, upon gaining dominance status, females, but less so males, show morphological and behavioural changes, such as increased aggression and increased body mass, helping them retain control over breeding opportunities^7^. In previous reproductive endocrine studies of meerkats, researchers identified a status-related difference in T, not in males^34^, but in pregnant females, specifically^7^. The generalisability of this difference outside of gestation, however, remains unknown. Likewise, assessment of heterologous hormones in males remains to be determined and the sexes have yet to be compared.

Our first aim was to characterise ‘baseline’ status and sex differences in the reproductive hormones of wild, sexually mature meerkats. We thus obtained blood and faecal samples from meerkats while they were outside of known reproductive events and while they were residing within their clan. For the first time in meerkats, we included measures of A_4_ because this steroid has been revelatory in other female-dominant species^12^,^16^,^17^. We assessed all serum samples using the same laboratory methods, performed over the same study period (see Methods), which allowed us to directly compare the sexes. Our second aim was to examine if reproductive variables further influenced any status-related endocrine patterns within the sexes. For males, the reproductive event we targeted was roving, which occurs when resident males temporarily leave their clan in pursuit of reproductive opportunities with extra-group females^35^,^36^. Researchers previously identified an increase in the T concentrations of subordinate males associated with roving^35^, but values for other sex steroids and for dominant males remain unknown. For females, the reproductive event we targeted was pregnancy. Researchers previously identified in dominant females, relative to subordinate females, greater E_2_ concentrations outside of gestation^31^,^34^,^37^ and greater T concentrations during pregnancy^7^. We aimed to complete comparisons of all three steroids, both during and outside of gestation. Because serum sampling was limited for animals during the targeted reproductive events, our final aim was to validate faecal analyses (against our serum analyses) to allow greater depth of monitoring.

## Results

### Baseline endocrine patterns

**Figure 1:**
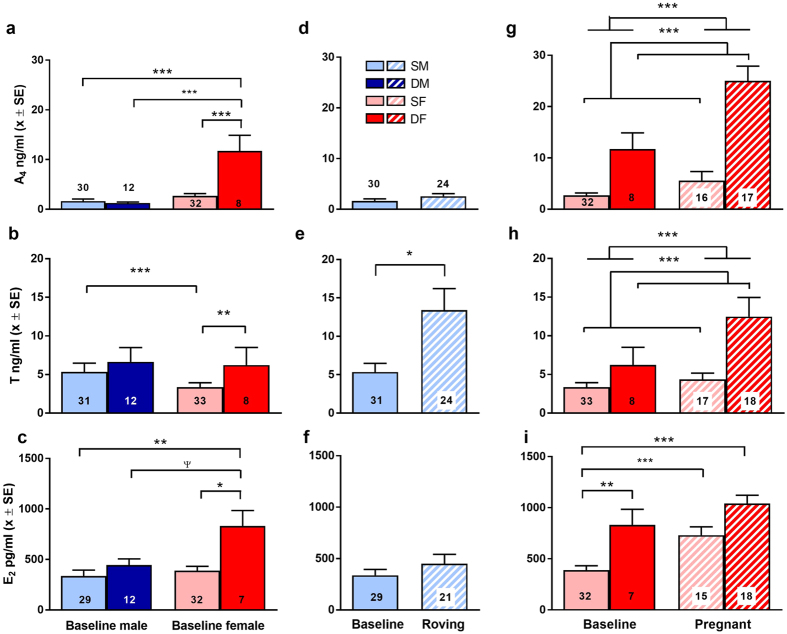
In sexually mature meerkats, dominant females (red) and animals during reproductive events (hatched bars) generally have greater concentrations of sex steroids than do either subordinate females (pink), dominant males (dark blue), and subordinate males (light blue) or animals outside of reproductive events (solid bars), respectively. Shown by sex and social status are mean + SEM baseline circulating concentrations of (top row) androstenedione (ng/ml), (middle row) testosterone (ng/ml) and (bottom row) estradiol (pg/ml). Shown by reproductive state for each steroid are (first column) baseline values (solid bars) for both sexes, (second column) subordinate male baseline values (solid bars) in relation to roving (hatched bars) and (third column) female baseline values (solid bars) in relation to pregnancy (hatched bars). Numbers of individuals are included for each category at the bottom of the bar graphs. ***P < 0.001, **P < 0.01, *P < 0.05 and ^ψ^P < 0.10.

In our study of serum endocrine patterns in meerkats, we found the interaction between sex and social status to be strongly predictive of variation outside of reproductive events (i.e. of baseline patterns for resident, non-roving males and resident, non-pregnant females: Table 1; Fig. 1; for sample sizes and other variables, see Supplementary Material). Dominant and subordinate males (DM and SM, respectively) showed no differences in serum concentrations of A_4_ (LSD: *t* = −0.699, *P* = 0.894; Fig. 1a), T (LSD: *t* = −0.906, *P* = 0.796; Fig. 1b) or E_2_ (LSD: *t* = −0.221, *P* = 0.996; Fig. 1c). By contrast, females showed strong status-related differences in all three of the sex steroids: dominant females (DF) had significantly greater serum concentrations of A_4_ (LSD: *t* = 6.628, *P* < 0.001; Fig. 1a), T (LSD: *t* = 3.927, *P* = 0.001; Fig. 1b) and E_2_ (LSD: *t* = 2.938, *P* = 0.023; Fig. 1c) than did subordinate females (SF).

Intersexual comparisons of these baseline endocrine patterns revealed even more striking patterns. Notably, the A_4_ concentrations of dominant females were not only elevated by comparison to other female mammals (see Table 2 in Drea 2007)^12^, including female-dominant species assayed in the same manner as in the present study^16^, but were significantly greater than those of conspecific males (LSD: DF vs. DM: *t* = 5.832, *P* < 0.001; DF vs. SM: *t* = 5.984, *P* < 0.001; Fig. 1a). Even the A_4_ concentrations of subordinate females were comparable to those of resident males (LSD: SF vs. DM: *t* = −0.613, *P* = 0.925; SF vs. SM: *t* = −0.134, *P* = 0.999; Fig. 1a). Moreover, dominant females had T concentrations equivalent to those of both classes of males (LSD: DF vs. DM: *t* = 1.209, *P* = 0.615; DF vs. SM: *t* = 0.493, *P* = 0.959; Fig. 1b). T concentrations in subordinate females, albeit significantly lower than those of subordinate males (LSD: *t* = −4.332, *P* < 0.001; Fig. 1b), did not differ significantly from those of dominant males (LSD: *t* = 2.064, *P* = 0.170; Fig. 1b). Lastly, the E_2_ concentrations of dominant females were significantly greater than those of subordinate males (LSD: *t* = 3.535, *P* = 0.004; Fig. 1c) and tended to be greater than those of dominant males (LSD: *t* = 2.529, *P* = 0.053; Fig. 1c), but the E_2_ concentrations of subordinate females were equivalent to those of males (LSD: SF vs. DM: *t* = −0.822, *P* = 0.838; SF vs. SM: *t* = −0.950, *P* = 0.770; Fig. 1c).

### Endocrine patterns during reproductive events

We confirmed that roving subordinate males have greater T concentrations than do non-roving subordinate males (GLMM; *P* = 0.03; Table 1; Fig. 1e); however, there appeared to be no relation, between roving and either serum concentrations of A_4_ (GLMM; *P* > 0.05; Table 1; Fig. 1d) or E_2_ (GLMM; *P* > 0.05; Table 1; Fig. 1f) in subordinate males. Obtaining serum samples from roving dominant males presented logistical challenges, such that we excluded dominant males from these analyses (but see below for results from faecal analyses). Nevertheless, based on a single blood sample obtained from a dominant male following copulation observed within the clan, serum T (67.4 ng/ml) was an order of magnitude greater than the mean baseline value. By contrast, concomitant A_4_ (5.4 ng/ml) and E_2_ (389.7 pg/ml) concentrations in this dominant male were well within normal ranges. To the extent that this example might be broadly representative, sexual activity, whether directly observed or implied from prospecting forays, led to substantial T increases in males of both social classes.

Gestation significantly increased both serum A_4_ and T concentrations across all females (GLMM; *P*s < 0.001; Table 1; Fig. 1g,h). Moreover, consistent with status effects observed outside of pregnancy, dominant dams had greater A_4_ and T concentrations than did subordinate dams (GLMM; *P*s < 0.001; Table 1; Fig. 1g,h). Based on these data, pregnant female meerkats (particularly dominant dams) arguably had greater T concentrations than resident, adult males (Fig. 1b,h), with pregnancy raising female T concentrations to values commensurate with those of roving subordinate males (Fig. 1e,h). With regard to E_2_ concentrations during gestation, we found a significant interaction between status and reproductive state. Although, in subordinate females, serum E_2_ concentrations were greater during gestation than during non-pregnancy (LSD: *t* = −4.911, *P* < 0.001; Fig. 1i), the same was not true for dominant females (LSD: *t* = −0.604, *P* = 0.927; Fig. 1i). Thus, the status effect on E_2_ concentrations observed outside of pregnancy was not maintained during gestation; instead, dominant and subordinate dams had equivalent E_2_ concentrations (LSD: *t* = −1.276, *P* = 0.571; Fig. 1i).

### Baseline and reproductive patterns in faecal androgen metabolites (FAM)

**Figure 2:**
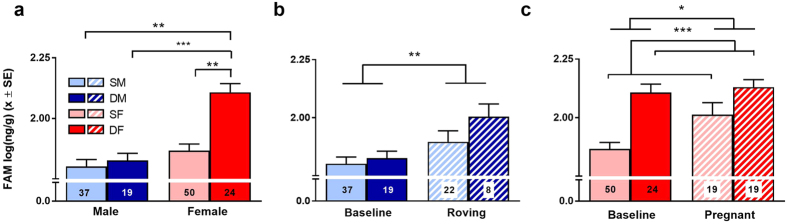
Concentrations of faecal androgen metabolites in sexually mature meerkats largely reproduce patterns observed for concentrations of serum androgens: dominant females (red) and animals during reproductive events (hatched bars) have greater concentrations than do either subordinate females (pink), dominant males (dark blue), and subordinate males (light blue) or animals outside of reproductive events (solid bars), respectively. Shown are mean + SEM logged concentrations of faecal androgen metabolites (ng/g) by (**a**) baseline sex (solid bars) and social status for both sexes, (**b**) male baseline values (solid bars) in relation to roving (hatched bars) and (**c**) female baseline values (solid bars) in relation to pregnancy (hatched bars). Numbers of individuals are included for each category in the bottom of the bar graphs. ***P < 0.001, **P < 0.01 and *P < 0.05.

We repeated the above analyses and comparisons using values of androgen metabolites derived from faecal samples, which allowed us to increase our sample sizes and include dominant male rovers (see Supplementary Tables S1 & S2). The effects of sex, social status, and reproductive events that we observed for serum androgens were appropriately captured by our analyses of FAM (Fig. 2; Supplementary Table S3; for other variables, see Supplementary Material). These findings replicate and extend our previous validations of faecal androgen assays in this species^38^. After controlling for the effects of age, we still observed the following: (1) the absence of male status differences (LSD: *t* = −0.939, *P* = 0.779; Fig. 2a), (2) the presence of female status differences both during baseline (LSD: *t* = 3.272 *P* = 0.007; Fig. 2a) and reproductive phases (LMM: *χ*^*2*^ = 11.609, *P* < 0.001; Fig. 2c), (3) the increased androgen concentrations of dominant females relative to males of either social class (LSD: DF vs. DM: *t* = 4.827, *P* < 0.001; DF vs. SM: *t* = 3.601, *P* = 0.003; Fig. 2a), (4) the equivalence of androgen concentrations between subordinate females and males of either social class (LSD: DM vs. SF: *t* = −1.933, *P* = 0.215; SF vs. SM: *t* = 1.081, *P* = 0.696; Fig. 2a), (5) the increase in androgens with male roving (in this case, in all males: LMM: *χ*^*2*^ = 7.811, *P* = 0.005; Fig. 2b) and (6) the increase in androgens with female pregnancy (LMM: *χ*^*2*^ = 4.397, *P* = 0.036; Fig. 2c).

## Discussion

Focusing on the endocrine correlates of heightened aggression and reproductive competition in females, we compared sex steroid concentrations of male and female meerkats, across baseline and reproductive phases. Based on these comparisons, we report on exceptional endocrine patterns in both sexes, including evidence of increased estrogen concentrations in males (relative, minimally, to conspecific subordinate females) and, more unusually, of substantially increased androgen concentrations in females (relative, minimally, to conspecific males). Whereas estrogens in male meerkats may be associated with their predisposition for infant care (given that all males of this species, albeit to varying degrees, engage in babysitting or pup feeding behaviour^39^), androgens in females likely underlie their pronounced aggressiveness. Within females (but not within males), dominant individuals had greater concentrations of sex steroids than did their subordinate counterparts, suggesting that female social status may be hormonally mediated in a manner that male social status is not. Such an interpretation may provide a proximate mechanism to explain the behavioural and morphological changes uniquely observed in female meerkats upon dominance acquisition^7^,^37^. Additionally, gestation magnified the effects of social status on female endocrine values, potentially affording dominant dams an additional competitive edge at a time when access to food resources is critical and threat of infanticide is high. Lastly, as shown or suggested for other ‘masculinised’ species^16^,^19^,^27^ and for certain clinical cases of ‘androgenised’ women^40^,^41^, increased gestational A_4_, coupled with appropriate placental conversion to T, may provide a mechanism of ‘inheritance’ to influence the development of meerkat daughters. The present data provide evidence of an unusually extreme form of female hormonal masculinisation that may underlie female aggression and reproductive skew in this species.

In meerkats, dominant and subordinate males generally had comparable endocrine values during reproductive and non-reproductive periods, respectively. In particular, the equivalence of T by male social status reported herein was consistent with a prior study in meerkats^34^. Although this pattern differs from that observed in cooperative breeders in which adult subordinate males are physiologically suppressed from reproducing^21^,^42^, it is not unusual in males of all cooperatively breeding species^43^,^44^. In meerkats, some of the reproductive skew observed between males might be attributed to incest avoidance^32^. In addition, as has been suggested for cooperatively breeding primates^45^, the dominant male’s behavioural ‘suppression’ of subordinates, through mate guarding, may reduce his need to ensure paternity via status-related hormonal suppression. The present findings are consistent with a role for T in the reproductive activity, but not the intrasexual social status, of male meerkats.

With regard to intersexual social status, it may be relevant that the traditional female bias in E_2_ was less pronounced in adult meerkats than is typical of most mammals: male meerkats had relatively raised E_2_ concentrations (i.e., comparable to those of subordinate female meerkats), consistent with patterns detected in the males of some other female-dominant species, such as binturongs and lemurs^29^,^30^. Although circulating concentrations of E_2_ in males (when even detectable) are less often measured than they are in females^46^, E_2_ can have important functions in adult male mammals, including in the mediation of sexual function^47^,^48^ and the expression of paternal behaviour^46^. Given the concentrations we detected, perhaps E_2_ may facilitate male social deference or male infant care in meerkats. Further investigation should be aimed at better understanding the role of E_2_ in male mammals more generally^29^,^46^.

In female meerkats, we found that the status-related difference in T previously reported during pregnancy^7^ was not limited to gestation, but characterised females year round. Thus, unusual T concentrations in pregnant female meerkats do not owe solely to the physiology of gestation, per se, nor to the status-related behavioural differences accompanying gestation (i.e., the dominant dam’s increased aggressive targeting of subordinate dams^49^) that might raise her T concentrations. Instead, there appears to be a basic biological difference between the social statuses (or breeding classes) that transcends the females’ reproductive cycles and exists beyond short-term periods of endocrine activation. When access to resources important for reproductive success are limited, selection for traits that can increase an individual’s competitive abilities may be maximised^25^,^50^, which may be reflected, in female meerkats, by pervasive status-related differences in the hormones mediating aggression.

The critical mediating hormones, in this case, may involve both A_4_ and T. The female bias in A_4_ concentrations we observed in meerkats also characterises the spotted hyaena^17^,^51^; otherwise, a sex reversal in A_4_ or even an equivalence in A_4_ between the sexes is rare (if not absent) in other mammals^12^,^51^,^52^. Moreover, meerkats appear to be characterised by the general absence of a sex difference in T – a pattern that is extremely unusual among mammals^53^. Only in the rock hyrax (*Procavia capensis*) has there been a report of a sex reversal in mammalian T concentrations^54^; however, among female hyraxes, rank is curiously, negatively correlated to T^55^. Otherwise, even in the most masculinised of females, T concentrations outside of pregnancy fall well below those of conspecific males^12^,^24^. Even the traditional male bias in T concentrations we found between subordinate meerkats was reduced by comparison with other mammals (see Table 2 in Drea 2007 for blood concentrations of T and A_4_ in adult male and nonpregnant female mammals)^12^. The source of these androgens, however, remains unknown. A_4_ in female meerkats, as in female spotted hyaenas, might be an important precursor to T. Unlike the spotted hyaena, however, in which raised T concentrations are confined to gestation (owing to placental conversion from A_4_ via 17ß-hydroxysteroid dehydrogenase)^17^,^18^,^19^, the substantial T concentrations we observed in meerkats year round may indicate that A_4_ is biologically available for conversion to T outside of pregnancy. Therefore, T in female meerkats could readily explain female aggressiveness in adulthood; it might also be transferred to developing foetuses during gestation and provide a mechanism to behaviourally masculinise daughters. Future research will be aimed at testing the hypothesis of female behavioural masculinisation through manipulation of androgens in pregnancy. It also would be interesting to examine 17ß-hydroxysteroid dehydrogenase activity in the meerkat, as well as potential mechanisms used to tune the window of prenatal exposure to androgens. The latter presumably prevent female reproductive problems that could be otherwise encountered^56^, including as a result of genital masculinisation^19^.

Despite the potential benefits for intrasexual selection of hormonal masculinisation in a female-dominant species, increased female androgen concentrations and female dominance in other species do not come without direct costs^57^, such as reduced reproductive success^58^,^59^, reduced offspring care^60^ and increased risks from aggression^61^. Despite the indirect costs on subordinate reproduction (through increased resource competition, eviction and infanticide by dominant females), there is currently no evidence that these same direct, reproductive costs occur in the cooperatively breeding meerkat: Although a potential androgen-mediated reduction in offspring care might be masked by the cooperative nature of this species, dominant females breed successfully and at higher rates than do subordinates. Another direct cost, however, namely androgen-mediated immunosuppression^62^, but see ref. 63, may occur via female-biased parasitism, which is especially pronounced in dominant females^64^,^65^. Nevertheless, the advantages gained by increased survival and reproductive success likely outweigh any direct cost of increased female androgen concentrations. Together, our findings are consistent with hormonal masculinisation of the female meerkat – masculinisation that is greater in dominant than in subordinate females – and consistent with suggestions of intrasexual selection operating in females^7^.

## Methods

### Study site and subjects

Our study was conducted, between November 2011 and April 2015, on a habituated, wild population of meerkats inhabiting the Kuruman River Reserve, South Africa (26°58′S, 29°49′E). Details on the study site, habitat and climate have been provided elsewhere^66^. Clan and population numbers fluctuated annually, with an annual average of roughly 270 individuals from 22 clans, and a total during our study period of roughly 810 animals from 43 clans. All of the animals were individually identifiable via unique dye marks and were habituated to close observation (<2 m) and routine weighing^37^. The clans were visited and observed at least every three days. Thus, we knew the social status of each animal and could collect detailed life history, weight and endocrine data (see below).

Our focal subjects were the sexually mature members of 23 of the clans, including 93 males (70 subordinate, 32 dominant) and 91 females (69 subordinate, 35 dominant), aged 0.75–9.4 years (mean + S.E.M.: 2.6 + 0.07; see Supplementary Tables S1 & S2). The inclusion of animals 9–12 months of age did not alter any of the results obtained when considering only animals 1 year or older (data not shown). We classified males as roving if they had been absent from the group (either on their own or in a coalition with other males) in the two days preceding or following sample collection (see below). We could detect pregnancy at around 3–4 weeks of gestation by an increase in the dam’s weight and by visible swelling of her belly. Because meerkat gestation lasts 70 days^67^, we estimated the date of conception by subtracting 70 days from the known date of birth. We included only samples from pregnancies that resulted in a successful birth (i.e., we excluded those from pregnancies resulting in abortion). We also excluded samples collected during the first week post-partum, to avoid any potential carry-over effect of gestation on sex steroids. Within sexes and social classes, we sampled both from different individuals and from the same animals across different conditions (e.g. outside of and during pregnancy). Accordingly, roughly 30% of individuals were repeated across treatments and roughly 67% of individuals were sampled more than once.

Our protocols were approved by the Institutional Animal Care and Use Committee of Duke University (Protocol Registry Numbers A171-09-06 and A143-12-05) and by the University of Pretoria’s Animal Use and Care Committee (Ethical Approval Number EC074-11). Our methods were carried out in accordance with the approved guidelines.

### Sampling procedures

We individually captured and processed the animals to minimise the time delay (mean ± S.E.M. = 8.11 ± 0.23 min) until blood draw. We gently picked up the subjects, by the tail base, carefully placed them into a cotton sack and anaesthetised them with isoflurane (Isofor; Safe Line Pharmaceuticals, Johannesburg, South Africa) in oxygen, using a vehicle-mounted vaporiser^68^. Using a 25 G needle and syringe, we drew 0.2–2 ml of blood from the jugular vein. We allowed blood samples to clot at ambient temperature in serum separator tubes (Vacutainer^®^, Becton Dickinson, Franklin Lakes, NJ, USA), then centrifuged them at 3700 rpm, at 24 °C, for 10 min. We stored the decanted serum samples on site at −40 °C until transport, on ice, to Duke University in North Carolina, where we kept them at −80 °C until assay. Of the 223 blood samples, 216 (97%) were obtained in the morning and 7 (3%) in the afternoon. Steroid values derived from afternoon samples, obtained from all subject categories except subordinate females, fell well within the distribution of values derived from the morning samples of their respective cohorts (see also Analyses).

Faecal sampling occurred ad libitum, throughout the day, during routine observational periods. We collected the fresh samples in clean plastic bags and immediately placed them on ice. Upon return from the field, we stored the samples, on site, at −40 °C until transport, on ice, to Duke University, where we kept them at −80 °C until assay. We previously could detect no diurnal effect on the concentration of androgen metabolites derived from faecal samples^38^.

### Hormone assays

We determined serum concentrations of A_4_, T and E_2_ using commercial, competitive enzyme immunoassay (EIA) kits (ALPCO diagnostics, Salem, NH, USA). We validated the EIA serum assays by standard parallelism, linearity and recovery tests^69^. Samples with concentrations greater than the upper detection limit were diluted with assay buffer to no more than 1:8, and the results obtained were then multiplied by the dilution factor. Samples that had concentrations below the minimum detectable limit of the assay were allocated this minimum value. All samples were run in duplicate and were re-run in a subsequent assay if the coefficient of variation (CV) exceeded 10%. Capture to bleed time was recorded for all samples and found to be non-significant for A_4_, T and E_2_ (ANOVA: *P*_1_ = 0.867, 0.192 and 0.724, respectively).

The A_4_ assay has a sensitivity of 0.04 ng/ml using a 25-μl dose, with an intra- and inter-assay CV of 5.23% and 8.7%, respectively. Serial dilutions of pooled meerkat serum yielded a displacement curve parallel to the A_4_ standard curve. Assay accuracy, measured as percent recovery of known amounts of analyte from a pooled serum sample was 100.3% (n = 6). Cross reactivity of the A_4_ assay was 1.8% with dehydroepiandrosterone (DHEA), 0.2% with T, <0.1% with estrone, E_2_, progesterone, 17-OH progesterone and 5α-dihydrotestosterone (DHT), <0.01% with cortisol and DHEA sulphate (DHEA-S). The T assay has a sensitivity of 0.02 ng/ml using a 50-μl dose, with an intra- and inter-assay CV of 7.9% and 7.3%, respectively. Serial dilutions of pooled meerkat serum yielded a displacement curve parallel to the T standard. Accuracy was 110% (n = 6). Cross reactivity of the T assay was 5.2% with DHT, 1.4% with A_4_, 0.8% with androstanediol, 0.5% with progesterone, 0.1% with androsterone and <0.1% with aldosterone, andrenosterone, cholesterol, corticosterone, DHEA, DHEA-S, epiandrosterone, E_2_, estriol and pregnenolone. The E_2_ assay has a sensitivity of 10 pg/ml using a 50-μl dose, with an intra- and inter-assay CV of 7.7% and 8.7%, respectively. Serial dilutions of pooled meerkat serum yielded a displacement curve parallel to the E_2_ standard curve. Accuracy was 104.2% (n = 6). Cross reactivity of the E_2_ assay was 1.6% with estriol, 1.3% with estrone, and 0.1% with progesterone and cortisol.

We extracted faecal androgen metabolites (FAM) and assayed them via EIA using protocols previously validated for male meerkats^38^. Here, we extend the previous validation by comparing FAM patterns with circulating concentrations of androgens (A_4_ and T). Assay sensitivity was 0.2–12.5 ng/ml per plate, with an intra- and inter-assay CV of 7.7% and 6.2%, respectively. Cross reactivity of the FAM assay was 100% with T, 9% with DHT, <1% with androstenediol, and <0.1% with A_4_, estriol, E_2_ and progesterone.

### Statistical analyses

We analysed serum A_4_, T and E_2_ concentrations using generalised linear mixed models (GLMMs) in R 3.2.2^70^ and the MASS package (version 7.3–43). We analysed response variables using a Gamma error distribution and log link function, and included individual identity as a random factor to account for repeated sampling of individuals. We initially included all probable independent terms and interactions (assessed via variance inflation factors) in the full model; we then obtained a minimal model by sequential removal of the least significant factors (*P* < 0.05), starting with two-way interactions. We determined significance of fixed factors through maximum likelihood estimation and likelihood ratio tests following a χ^2^ distribution. We confirmed validity of the minimal model using a forwards stepwise procedure^71^. We verified that all model assumptions were met by checking residuals from both full and minimal models for normality and homogeneity of variance. We compared significant interactions using post hoc pairwise comparisons (LSD) in the lsmeans package (version 2.21). Owing to occasional small serum volumes, we could not analyse all three steroids in all of the samples. In these cases, we prioritized analysing either A_4_ or T over E_2_ (see Supplementary Table S1). All statistical tests were two-tailed and, unless otherwise stated, we present means ± SEM.

To test for sex and status differences in baseline sex steroid concentrations, we used a total of 145 samples collected from 87 individuals (see Supplementary Table S1) outside of reproductive events (defined as follows: for males, we excluded samples taken within two days of roving; for females, we excluded samples taken during gestation and the first week post-partum). We included as fixed factors in the full model an interaction between social status (dominant or subordinate) and sex (female or male), as well as an individual’s age (in months) at sample collection, mean weight (in g) for the 30 days preceding sample collection and total rainfall (in mm) for the 30 days preceding sample collection.

To test if reproductive events related to sex steroid concentrations in subordinate males, we used a total of 85 samples from 47 individuals (we lacked samples to conduct this analysis in dominant male rovers; see Supplementary Table S1). We included reproductive state (baseline or roving), age, mean weight and total rainfall as fixed factors in the full model. To test if reproductive events related to sex steroid concentrations in females, both in dominant and subordinate animals, we used a total of 121 samples from 61 individuals (see Supplementary Table S1). We included as fixed factors in the full model an interaction between reproductive state (baseline or pregnant) and social status, as well as age and total rainfall. We did not include weight as a covariate in this model due to its collinearity with pregnancy state.

We analysed FAM concentrations using linear mixed models (LMMs) in R 3.2.2^70^ using the lme4 package (version 1.1–10). After log transformation, response variables conformed to normal distribution and so we analysed them using a gaussian error distribution and identity link function, and included individual identity as a random factor to account for repeated sampling of individuals. We initially included all probable independent terms and interactions (assessed via variance inflation factors) in the full model; we then obtained a minimal model, by sequentially removing terms based on the Akaike information criterion (AIC). We confirmed validity of the final model using a forward stepwise procedure^71^. We determined significance of fixed factors through maximum likelihood estimation and likelihood ratio tests following a χ^2^ distribution. We verified that all model assumptions were met by checking residuals from both full and minimal models for normality and homogeneity of variance. We compared significant interactions using post hoc tests in the lsmeans package (version 2.21).

To test for sex and status differences in baseline FAM concentrations, we used a total of 341 samples collected from 130 individuals outside of reproductive events (as previously defined; see Supplementary Table S2). We included an interaction between social status and sex, as well as age, mean weight, total rainfall, and collection time (AM or PM) as fixed factors in the full model.

To test if reproductive events related to male FAM concentrations in both dominant and subordinate animals, we used a total of 193 samples from 66 individuals (see Supplementary Table S2). We included an interaction between reproductive state and social status, age, mean weight, total rainfall and collection time as fixed factors in the full model. To test if reproductive events related to female FAM concentrations in both dominant and subordinate animals, we used a total of 306 samples from 78 individuals (see Supplementary Table S2). We included an interaction between reproductive state and social status, as well as female lactation state (lactating or not lactating), age, total rainfall, and collection time as fixed factors in the full model. Again, we did not include weight as a covariate in this model due to its collinearity with pregnancy state.

## Additional Information

**How to cite this article**: Davies, C. S. *et al*. Exceptional endocrine profiles characterise the meerkat: sex, status, and reproductive patterns. *Sci. Rep.* **6**, 35492; doi: 10.1038/srep35492 (2016).

## Supplementary Material

Supplementary Information

## Figures and Tables

**Table 1:** Factors associated with reproductive hormones during ‘baseline’ and reproductive events in wild meerkats.

	Model terms	Androstenedione			Testosterone			Estradiol		
		Estimate (SE)	χ2	P	Estimate (SE)	χ2	P	Estimate (SE)	χ2	P
Baseline	Status	−2.67 (0.41)	17.9	<0.001	−2.02 (0.51)	5.34	0.02	−0.98 (0.31)	6.22	0.005
	Sex	−2.95 (0.51)	6.07	0.01	−0.84 (0.68)	10.69	0.001	−0.99 (0.38)	3.4	0.003
	Average weight	0.003 (0.001)	8.33	0.004	0.007 (0.001)	30.34	<0.001	0.005 (0.001)	30.49	<0.001
	Rainfall	—	—	—	−0.18 (0.009)	4.17	0.04	—	—	—
	Age	—	—	—	—	—	—	−0.02 (0.008)	4.11	0.04
	Status*Sex	2.98 (0.56)	29.73	<0.001	2.62 (0.75)	12.76	<0.001	0.83 (0.42)	4.18	0.04
Roving	Average weight	0.005 (0.001)	20.28	<0.001	0.006 (0.001)	17.84	<0.001	0.004 (0.001)	15.16	<0.001
	Roving	—	—	—	0.59 (0.27)	4.85	0.03	—	—	—
Pregnancy	Status	−1.92 (0.27)	53.96	<0.001	−1.37 (0.35)	16.02	<0.001	−0.89 (0.24)	16.66	<0.001
	Pregnant	0.97 (0.21)	21.36	<0.001	0.97 (0.22)	19.56	<0.001	0.16 (0.26)	24.22	<0.001
	Status*Pregnant	—	—	—	—	—	—	0.64 (0.31)	5.14	0.02

Random effects = Individual.

All comparisons made against the indicated levels of each factor (status = dominant, sex = female, reproductive state = baseline).

χ2 = likelihood ratio test statistic; df = 1.
