# Supplementary material for: Exceptional endocrine profiles characterise the meerkat: sex, status, and reproductive patterns
Source: Sci Rep. 2016 Oct 18;6:35492. doi: 10.1038/srep35492 (PMC5067592; doi:10.1038/srep35492)
Supplement: Supplementary Information [file srep35492-s1.pdf]

Exceptional endocrine profiles characterise the meerkat: sex, status, and reproductive patterns

Charli S. Davies[1,2], Kendra N. Smyth[1,2,3], Lydia K. Greene[1,2,3], Debbie A. Walsh[1,2], Jessica Mitchell[1,2], Marta B. Manser[1,4,5], Tim Clutton-Brock[1,5,6], and Christine M. Drea*,[1,2,3,7]

[1]Kalahari Research Trust, Kuruman River Reserve, Northern Cape, South Africa

[2]Department of Evolutionary Anthropology, Duke University, Durham, NC 27708 USA

[3]University Program in Ecology, Duke University, Durham, NC 27708 USA

[4]Institute of Evolutionary Biology and Environmental Studies, University of Zurich, Zurich, Switzerland

[5] Mammal Research Institute, University of Pretoria, Pretoria, South Africa

[6]Department of Zoology, University of Cambridge, Cambridge, UK

[7]Department of Biology, Duke University, Durham, USA

*Author for correspondence:

Christine M. Drea, e-mail: cdrea@duke.edu

Supplementary Information for Serum Endocrine Samples

Table S1. Numbers of subjects and sample sizes for serum analyses

	All Samples		Androstenedione		Testosterone		Estradiol	
	Animals	Samples	Animals	Samples	Animals	Samples	Animals	Samples
**Females - total**	58	121	56	116	57	116	55	108
Dominant - baseline	8	9	8	9	8	9	7	8
Dominant - pregnant	18	27	17	26	18	27	18	26
Subordinate - baseline	35	63	32	60	33	59	32	55
Subordinate - pregnant	17	22	16	21	17	21	15	19
**Males - total**	57	102	56	98	57	99	52	87
Dominant - baseline	12	17	12	17	12	17	12	13
Subordinate - baseline	32	56	30	52	31	55	29	50
Subordinate - Roving	24	29	24	29	24	27	21	24
**OVERALL TOTAL**	115	223	112	214	114	215	107	195

Additional variables in relation to serum endocrine patterns. Beyond the interaction between sex and social status, weight was also a significant predictor of baseline, serum endocrine patterns. For instance, relative to light-weight meerkats, heavier animals showed slightly greater concentrations of A_4, T and E_2 (GLMM; $P < 0.005$, $P < 0.001$, $P < 0.001$, respectively; Table 1), although these findings could have owed to dominant individuals, and specifically dominant females, being heavier on average, than subordinates (ANOVA; $F_1 = 36.2$, $P < 0.001$), which is typical of meerkat society[1,2]. Other variables, including age and rainfall, explained little to none of the variation (Table 1).

Table S2. Numbers of subjects and sample sizes for faecal analyses

	Animals	Samples
**Females - total**	78	306
Dominant - baseline	24	59
Dominant - pregnant	19	69
Subordinate - baseline	50	137
Subordinate - pregnant	19	41
**Males - total**	66	193
Dominant - baseline	19	59
Dominant - roving	8	16
Subordinate - baseline	37	86
Subordinate - roving	22	32
**OVERALL TOTAL**	144	499

Additional variables in relation to faecal androgen metabolite patterns. As with the results derived from serum analyses, the interaction between sex and social status, explained most of the variation in baseline faecal androgen metabolites or FAM (Supplementary Table S3). Here, we found that age, rather than weight, was a modest, but significant, predictor of FAM concentrations, with older individuals of both sexes having greater concentrations both during baseline ($P = 0.009$; Table S3) and reproductive phases (males; $P = 0.046$, females; $P = 0.02$; Table S3). The effect of age can be partially explained by dominant individuals of both sexes being significantly older than subordinate individuals (ANOVA; $F_1 = 307.6$, $P < 0.001$), which is typical of meerkat society[1,2].

Table S3. Factors associated with faecal androgen metabolites during baseline and reproductive events in wild meerkats, with bolded font showing significant terms

	Model terms	Estimate (SE)	χ^2	*P*
**Baseline**	Status	-0.33 (0.14)	0.68	0.41
	**Sex**	**-0.61 (0.14)**	**12.96**	**<0.001**
	**Age**	**0.006 (0.002)**	**6.91**	**0.009**
	**Status*Sex**	**0.48 (0.17)**	**8.15**	**0.004**
**Roving**	**Roving**	**0.27 (0.10)**	**8.14**	**0.004**
	**Age**	**0.004 (0.002)**	**3.97**	**0.046**
	AM.PM	-0.15 (0.09)	2.93	0.087
**Pregnancy**	**Status**	**-0.28 (0.15)**	**1.81**	**0.19**
	**Pregnancy**	**0.09 (0.12)**	**5.41**	**0.02**
	**Age**	**0.006 (0.002)**	**5.28**	**0.02**
	Rainfall	0.003 (0.001)	3.18	0.07
	Status*Pregnancy	0.25 (0.17)	2.01	0.16

Random effects=Individual.

All comparisons made against the indicated levels of each factor (status = dominant, sex = female, reproductive state=baseline).

$\chi 2$ = likelihood ratio test statistic; df = 1.

[1] Clutton-Brock, T. H. *et al.* Cooperation, control, and concession in meerkat groups. *Science* **291**, 478-481 (2001).

[2] Clutton-Brock, T. H. *et al.* Intrasexual competition and sexual selection in cooperative mammals. *Nature* **444**, 1065-1068 (2006).

Table S4. Raw serum data

Sample number	Subject	Sex	Rank	Breeding	Age (mo)	Mean weight	Total rain (mm/mo)	Capture to bleed (min)	E2 (pg/ml)	A4 (ng/ml)	T (ng/ml)
6920	VJXM066	M	D	No	48.0	779.00	5.40	7	688.53	3.30	12.46
6990	VLM210	M	S	No	12.0	530.00	2.00	8	151.60	0.23	0.39
3268	VRRF116	F	D	Yes	49.6	791.13	9.00	6	550.25	50.68	11.06
3846	VWF147	F	D	Yes	49.5	638.00	41.80	9	1394.11	18.56	11.30
3586	VRRF137	F	D	Yes	33.2	813.95	0.00	7	na	8.70	7.62
5857	VJXF058	F	D	Yes	23.7	636.67	1.60	3	832.65	11.32	0.61
6982	VKUM070	M	D	No	12.0	545.11	2.40	10	417.86	0.97	11.18
3262	VAZM051	M	S	Yes	8.8	470.89	9.00	10	64.27	0.41	0.91
5780	VUKF003	F	D	Yes	24.3	646.20	1.60	3	1826.88	na	57.17
6753	VBBM091	M	S	No	12.0	665.65	4.40	9	259.87	1.62	13.80
3790	VSQF012	F	D	Yes	51.3	824.57	35.00	5	1110.62	8.17	0.69
3671	VBBF069	F	D	Yes	33.9	763.08	5.00	7	1035.49	25.78	13.29
3302	VLF102	F	D	Yes	97.6	791.00	9.00	7	730.70	19.59	1.96
3313	VSQM042	M	S	No	11.9	530.67	6.40	5	na	na	0.41
3240	VSQF012	F	D	Yes	37.9	890.42	12.20	11	576.49	29.87	2.39
6428	VJXM075	M	S	No	9.0	556.47	14.80	10	1102.90	6.61	26.15
2960	VGGF014	F	D	Yes	97.1	883.92	26.00	8	1250.75	23.74	4.82
3693	VRRF137	F	D	Yes	34.5	807.00	6.00	4	1206.91	47.74	31.60
3311	VSQM040	M	S	No	11.9	473.89	6.40	8	10.00	0.27	1.19
6415	VJXM073	M	S	Yes	8.9	530.31	14.80	9	1892.06	7.87	27.64
7154	VBBM080	M	D	No	38.0	737.60	4.60	2	na	0.88	1.57
3580	VKUF005	F	D	Yes	69.0	811.17	0.00	14	919.03	16.49	1.74
3837	VKUF005	F	D	Yes	71.5	854.75	42.00	11	790.03	15.55	2.11
7262	VLM155	M	S	No	72.1	613.50	18.20	7	133.94	0.65	0.79
6959	VVHM071	M	S	No	12.0	577.64	5.40	5	626.39	5.27	11.02
3319	VSQF012	F	D	Yes	40.4	823.91	6.40	8	931.20	27.53	2.16
3320	VVHM060	M	S	No	12.2	429.33	6.40	8	127.81	0.45	1.87
2968	VDF115	F	D	Yes	65.1	783.50	57.40	8	1180.22	28.62	10.63
4014	VBBF069	F	D	Yes	36.7	718.00	38.00	6	2001.26	61.32	31.24
6989	VLM211	M	S	No	12.0	504.47	2.00	9	239.29	0.52	3.67
2969	VLF111	F	D	Yes	82.9	822.75	57.40	6	1576.41	52.90	21.91
2997	VWF133	F	D	Yes	40.3	872.00	60.60	8	1529.81	35.38	9.29
5990	VVHF035	F	D	Yes	48.7	699.50	1.60	8	1406.95	31.30	20.65
3256	VWM175	M	S	No	8.8	397.64	9.00	6	33.49	0.51	2.23
3210	VDF115	F	D	Yes	73.6	782.89	10.00	6	1189.30	29.09	7.10
5214	VMYF005	F	D	Yes	9.0	686.00	0.00	10	1301.98	14.41	24.58
6616	VBBM062	M	D	No	48.6	789.15	13.20	5	na	0.68	2.64
6410	VWM186	M	S	No	9.0	437.56	14.80	9	357.50	0.60	0.99
7034	VUKF007	F	D	Yes	18.0	649.44	4.60	6	516.49	5.86	9.32
7188	VKUF005	F	D	Yes	84.1	689.14	6.20	10	673.61	17.61	1.73
3330	VJXM054	M	S	No	13.1	541.25	2.60	4	162.90	0.33	0.63
3699	VAZF020	F	D	Yes	47.7	696.85	6.00	9	702.03	10.62	20.79
3303	VRRF116	F	D	Yes	50.7	835.60	9.00	6	527.80	26.70	13.49
3325	VBBM085	M	S	Yes	12.0	404.31	2.60	5	na	0.07	0.02
3282	VSQM037	M	S	No	12.0	505.29	7.80	6	na	0.44	1.39
7178	VLF152	F	D	Yes	71.9	628.08	4.60	5	600.39	14.80	1.18
3689	VVHF035	F	D	Yes	38.5	701.60	6.00	7	696.43	18.40	16.54
6120	VBBM025	M	D	No	68.0	759.41	1.60	8	364.52	0.92	4.33
6039	VBBM085	M	S	Yes	32.1	635.25	1.60	6	220.29	0.83	1.73
3556	VBBM084	M	S	No	20.0	524.82	0.00	14	206.43	1.08	6.34
7724	VWF177	F	D	No	35.9	608.93	19.00	3	337.19	8.80	1.03

6871	VBBM092	M	S	No	12.2	626.64	4.40	8	136.63	0.95	5.62
6268	VMYF005	F	D	No	12.2	553.50	5.00	12	1642.53	30.61	19.54
3321	VVHM056	M	S	No	12.2	485.42	6.40	4	102.96	0.17	0.74
6750	VBBM094	M	S	Yes	12.0	694.53	4.40	8	176.07	1.04	9.55
3312	VSQM039	M	S	No	11.9	473.89	6.40	4	na	0.09	0.02
4615	VKUF065	F	D	No	28.4	633.28	14.00	10	673.60	11.26	0.63
5356	VKUF065	F	D	No	31.3	573.31	0.20	15	292.60	3.48	1.33
3216	VFM184	M	S	No	9.0	367.33	10.00	5	na	0.34	0.59
7159	VBBM090	M	S	Yes	15.9	643.67	4.60	10	223.44	1.41	8.29
3669	VAZF029	F	D	No	35.8	760.88	0.00	10	718.99	4.32	0.38
7155	VBBM089	M	S	Yes	15.9	668.13	4.60	9	107.25	0.04	10.31
5908	VKUF077	F	D	No	8.9	527.13	1.60	10	877.25	7.82	7.57
3487	VSQF005	F	D	No	57.1	764.00	0.00	9	994.18	23.66	4.45
4447	VAZF036	F	D	No	38.4	725.17	5.80	9	na	3.29	5.98
5758	VKUF039	F	D	No	49.4	728.43	0.20	4	1105.57	12.44	15.10
7157	VBBF088	F	S	Yes	15.9	641.33	4.60	13	813.28	5.77	0.02
3348	VWM175	M	S	Yes	11.9	416.00	4.00	5	na	0.20	na
5925	VRRF159	F	S	Yes	12.0	676.33	1.60	3	474.11	2.61	8.43
3757	VEKF010	F	S	Yes	34.5	737.58	6.60	5	na	6.64	11.16
5820	VRRF156	F	S	Yes	24.0	654.56	1.60	4	745.06	2.38	na
6395	VLF184	F	S	Yes	42.4	656.06	14.80	8	754.30	1.60	0.59
3818	VRRF156	F	S	Yes	15.3	674.36	34.00	6	588.37	2.28	5.24
6135	VBBM062	M	D	No	47.1	805.71	1.60	4	na	1.34	4.74
3264	VAZM053	M	S	Yes	8.9	474.85	8.20	6	75.99	1.14	3.74
7438	VBBM085	M	S	No	36.1	667.14	20.20	7	151.85	0.49	0.75
6960	VVHF072	F	S	Yes	12.0	622.67	3.20	8	417.45	0.73	0.21
6287	VBBM066	M	S	Yes	45.3	699.00	5.00	4	384.97	0.66	2.10
6278	VBBM041	M	S	Yes	61.6	744.00	5.00	4	404.37	0.76	4.33
3560	VAZF052	F	S	Yes	18.9	657.33	0.00	10	395.00	1.26	1.31
3214	VFM187	M	S	No	8.9	335.10	10.00	9	45.83	0.27	1.00
3220	VSQM040	M	S	No	9.0	431.47	10.00	6	74.72	0.43	2.02
3577	VWF177	F	S	Yes	19.3	661.06	0.00	10	1159.29	27.15	8.42
6208	VBBF083	F	S	Yes	32.5	752.09	0.40	6	1179.08	6.87	3.90
7455	VWM186	M	S	No	12.0	450.25	17.00	7	317.08	2.81	9.02
7015	VBBF083	F	S	Yes	35.1	690.00	4.40	5	1789.83	30.72	10.97
3558	VAZF027	F	S	Yes	43.2	702.08	0.00	6	543.63	2.49	3.37
3326	VBBM084	M	S	No	12.0	420.25	2.60	9	10.00	0.14	0.02
7472	VJXM073	M	S	No	12.0	536.85	22.20	12	1640.89	17.54	33.93
7767	VBBF093	F	S	Yes	14.9	732.00	19.20	8	490.01	0.53	0.25
3488	VBBF083	F	S	Yes	18.8	578.00	0.00	7	399.65	8.39	1.68
6679	VBBF077	F	S	Yes	39.1	787.33	13.80	8	535.43	na	5.45
3893	VJXF052	F	S	Yes	24.1	692.50	41.60	9	423.31	1.50	3.10
3198	VSQM037	M	S	No	9.0	411.92	1.80	5	61.30	0.46	0.10
6210	VWF176	F	S	Yes	31.5	626.39	0.40	14	691.60	2.93	9.20
5673	VBBM091	M	S	No	9.0	611.58	0.00	9	38.42	1.32	6.33
7402	VJXF057	F	S	Yes	28.0	634.54	19.20	9	828.11	5.06	4.78
4017	VBBM085	M	S	No	24.2	586.18	39.40	7	278.61	0.48	2.46
6105	VJXF057	F	S	Yes	24.4	692.55	1.60	10	1070.45	4.16	6.05
3832	VJXF035	F	S	Yes	37.6	820.50	42.00	12	na	1.06	1.61
6215	VRRF156	F	S	Yes	25.2	676.05	0.40	4	na	1.15	5.61
3702	VKUF065	F	S	Yes	22.8	638.50	6.00	9	594.49	1.49	0.61
3659	VEKF010	F	S	No	33.6	658.40	0.00	10	456.95	na	1.07
3675	VDF156	F	S	No	32.9	681.80	5.00	5	252.49	0.37	0.17
3255	VWF177	F	S	No	8.8	428.29	9.00	4	30.39	0.08	0.02
5959	VKUM070	M	D	No	9.1	528.13	1.60	10	519.07	1.07	2.19
6756	VBBF093	F	S	No	12.0	672.00	4.40	9	107.35	0.14	0.03

5992	VLM211	M	S	No	9.0	506.80	1.60	8	394.50	0.78	1.75
6101	VLM185	M	S	Yes	41.4	713.88	1.60	3	648.72	2.12	7.90
3261	VWF176	F	S	No	8.9	435.63	9.00	6	103.63	1.84	1.82
6604	VUKF012	F	S	No	16.7	614.00	12.80	5	na	0.04	0.02
3579	VCVM001	M	S	Yes	57.7	516.00	0.00	7	na	5.32	24.05
6316	VEKF022	F	S	No	12.1	533.77	5.00	6	562.90	4.33	6.87
3849	VTYF042	F	S	No	24.1	542.00	41.80	8	287.47	0.25	0.02
4009	VBBM084	M	S	No	24.1	594.14	38.00	13	na	1.00	0.97
3673	VDF155	F	S	No	32.9	683.46	5.00	22	460.32	0.70	na
6270	VEKF021	F	S	No	12.0	501.00	5.00	8	1102.10	9.27	10.56
6315	VEKF023	F	S	No	12.0	493.23	5.00	13	na	1.38	7.78
3209	VJXF052	F	S	No	9.1	425.83	11.80	7	157.58	4.73	1.78
3263	VAZF052	F	S	No	8.8	466.78	9.00	6	86.56	0.09	0.02
5646	VBBM092	M	S	No	9.0	591.00	0.00	10	280.13	0.71	2.75
3361	VVHM016	M	S	No	53.1	675.58	79.40	5	239.77	na	0.36
3566	VBBM085	M	S	No	20.2	521.23	0.00	10	180.76	0.93	2.45
7470	VJXM075	M	S	No	12.0	584.46	22.20	8	1440.46	5.49	14.58
7275	VBBM076	M	D	No	40.9	539.33	19.20	6	na	0.69	0.67
3503	VSQM039	M	S	Yes	20.0	574.88	0.00	11	155.57	0.79	3.43
3324	VBBF083	F	S	No	11.9	424.21	4.20	6	116.68	0.32	0.02
5826	VVHM071	M	S	Yes	8.8	528.36	1.60	7	1208.55	5.45	11.45
5824	VKUM059	M	S	No	37.4	682.54	1.60	5	709.85	3.53	8.90
3506	VSQM042	M	S	No	20.1	582.86	0.00	11	324.71	1.58	6.07
3294	VKUF065	F	S	No	12.0	471.36	7.80	4	17.40	0.06	0.02
6298	VBBM064	M	S	No	47.6	620.33	5.00	8	339.36	0.52	1.78
5766	VBBM090	M	S	No	11.9	687.50	1.40	6	251.62	0.62	1.73
3219	VSQF041	F	S	No	9.0	431.33	10.00	5	119.48	1.18	1.50
7643	VEKF022	F	S	No	15.9	544.71	11.40	16	504.34	3.59	4.40
4011	VBBF083	F	S	No	24.1	607.17	38.00	3	719.14	8.52	6.46
5949	VWM139	M	D	No	69.1	825.22	1.60	4	431.08	2.10	8.56
6961	VVHF073	F	S	No	12.0	537.00	3.20	7	460.45	1.41	1.21
7526	VLF206	F	S	No	18.1	548.85	23.40	6	na	0.08	0.04
3562	VJXF057	F	S	No	12.2	462.25	0.00	14	110.60	0.08	0.06
7626	VEKF021	F	S	No	15.9	543.83	11.40	5	658.00	12.34	16.02
5991	VLM210	M	S	No	9.0	541.47	1.60	9	435.70	0.94	2.02
6095	VZUM006	M	S	Yes	22.6	697.33	1.60	13	1017.56	8.04	59.62
5750	VLM177	M	S	Yes	46.6	727.80	0.20	6	1101.82	4.24	26.87
3543	VJXF058	F	S	No	12.0	441.00	0.00	14	421.02	0.66	0.02
3194	VTYF042	F	S	No	8.9	410.25	1.80	6	na	0.19	0.02
6958	VVHF070	F	S	No	12.0	613.64	5.40	6	646.79	3.83	2.99
5647	VBBF093	F	S	No	9.0	623.54	0.00	11	310.48	0.98	0.18
3502	VSQM040	M	S	No	20.0	519.84	0.00	11	228.22	0.94	5.17
5763	VBBM089	M	S	No	11.9	716.21	1.40	7	290.91	0.99	11.42
3505	VVHM060	M	S	No	20.0	485.36	0.00	11	271.64	0.97	6.52
5840	VVHF070	F	S	No	8.8	581.45	1.60	10	658.77	2.11	3.03
3500	VBBM042	M	S	Yes	48.2	707.92	0.00	10	na	1.09	8.87
4073	VWF176	F	S	No	23.9	750.20	39.60	9	299.77	3.54	3.13
5848	VEKF016	F	S	No	24.1	514.45	1.60	3	na	0.17	0.02
5957	VWM132	M	S	Yes	72.2	673.00	1.60	8	423.09	4.35	24.86
5752	VLM181	M	S	Yes	44.0	699.71	0.20	5	157.44	2.20	23.86
5827	VVHF072	F	S	No	8.8	556.55	1.60	7	375.90	0.25	0.15
7625	VEKF023	F	S	No	15.9	540.89	11.40	11	600.78	3.56	18.93
6212	VLM157	M	D	No	61.5	711.12	0.40	4	402.71	0.32	1.25
3383	VJXM055	M	S	Yes	16.1	549.91	0.00	3	330.08	3.97	7.84
4771	VBBM084	M	S	Yes	28.0	689.30	8.80	13	284.56	3.27	18.10
3376	VSQM037	M	S	No	16.0	579.83	0.00	5	56.39	0.54	1.36

3345	VWF176	F	S	No	11.8	442.46	4.00	9	552.67	12.42	10.68
3440	VWM175	M	S	Yes	16.1	500.06	15.40	11	102.53	0.54	1.96
4937	VBBM090	M	S	No	9.0	652.50	0.20	12	120.36	0.59	2.10
3215	VFF186	F	S	No	8.9	326.70	10.00	9	185.63	0.42	0.19
5769	VBBF088	F	S	No	11.9	661.86	1.40	7	216.27	0.08	0.02
6050	VUKF007	F	S	No	15.0	628.47	1.60	6	1690.37	12.04	10.54
6311	VBBM078	M	S	No	38.0	624.50	5.00	8	265.37	0.54	1.72
3547	VJXF035	F	S	No	34.7	644.55	0.00	na	na	2.58	na
3416	VSQM039	M	S	No	16.1	565.57	0.00	4	543.57	0.47	1.31
7085	VPAF016	F	S	No	15.6	566.59	4.60	3	401.62	0.24	na
5771	VBBM080	M	S	Yes	34.0	789.00	1.40	4	444.93	1.39	2.70
5754	VLM179	M	D	No	44.0	713.85	0.20	5	349.10	1.63	4.18
3504	VVHM056	M	S	No	20.0	547.10	0.00	9	227.33	0.44	2.23
3352	VAZF052	F	S	No	12.0	509.33	3.60	5	116.03	1.16	na
3574	VDF155	F	S	No	31.7	644.42	0.00	6	na	na	1.24
3421	VBBM085	M	S	No	16.0	494.13	0.00	5	567.43	na	na
7390	VJXF035	F	S	No	50.7	701.00	19.20	3	547.32	0.44	1.27
3381	VKUF065	F	S	No	16.0	492.73	0.00	4	25.23	0.10	0.02
3493	VJXF052	F	S	No	20.1	581.44	0.00	15	772.14	4.51	7.42
5225	VEKF021	F	S	No	9.0	480.64	0.00	11	949.87	10.38	14.02
6030	VBBF077	F	S	No	37.2	679.56	1.60	6	378.55	1.78	3.03
3439	VWF176	F	S	No	16.0	543.61	15.40	6	414.45	9.38	7.67
3417	VSQM040	M	S	No	16.2	527.10	0.00	4	10.00	0.08	0.02
3680	VKUF042	F	S	No	37.1	725.80	5.80	8	409.07	na	1.18
5756	VLM151	M	S	Yes	67.8	720.07	0.20	4	349.99	1.43	2.89
3382	VJXF052	F	S	No	16.1	531.09	0.00	7	323.32	2.97	10.62
4769	VBBM085	M	S	Yes	28.0	685.64	8.80	13	360.97	0.76	3.00
3422	VBBM084	M	S	No	16.0	498.60	0.00	4	104.94	0.32	1.39
4938	VBBM089	M	S	Yes	9.0	699.82	0.20	11	79.27	0.42	na
3446	VAZM051	M	S	Yes	16.0	547.86	15.40	8	577.07	12.29	47.28
5226	VEKF023	F	S	No	9.0	478.83	0.00	12	266.26	4.00	5.47
4298	VAZF052	F	S	No	25.4	692.00	0.40	12	374.72	2.26	3.28
3418	VSQM042	M	S	No	16.2	583.65	0.00	5	99.46	0.55	0.75
3885	VLM189	M	S	Yes	28.6	666.00	41.60	5	na	2.02	18.55
5227	VEKF022	F	S	No	9.0	483.18	0.00	8	111.66	2.38	2.30
5839	VVHF073	F	S	No	8.8	499.36	1.60	11	394.14	0.71	0.57
3419	VVHM056	M	S	No	16.3	530.33	0.00	4	45.27	na	1.53
5720	VUKF012	F	S	No	14.1	541.67	0.00	8	294.93	0.36	0.02
3445	VAZF052	F	S	No	16.0	582.93	15.40	7	233.76	0.83	1.34
3420	VVHM060	M	S	No	16.3	481.11	0.00	3	10.00	0.27	0.62
5048	VWF176	F	S	No	28.0	674.93	0.00	17	na	5.57	11.09
3882	VBBM042	M	D	No	52.0	777.91	41.60	11	158.26	0.47	2.68
3429	VJXF058	F	S	No	9.0	381.50	15.40	4	52.89	0.48	0.02
7627	VKUM070	M	D	No	13.9	494.75	11.40	11	348.33	0.46	2.22
4939	VBBF088	F	S	No	9.0	668.47	0.20	11	193.66	0.36	0.08
7354	VBBM025	M	D	No	71.4	698.07	19.20	2	161.30	0.60	3.84
3423	VBBF083	F	S	No	16.1	504.70	0.00	5	215.49	1.43	0.85
3428	VJXF057	F	S	No	9.0	391.50	15.40	10	67.80	0.67	0.02
5913	VVHM038	M	D	No	45.9	608.53	1.60	8	303.55	0.85	4.02
6056	VPAM014	M	D	No	45.5	747.00	1.60	3	712.85	2.46	31.12
3461	VDF166	F	S	No	8.8	465.62	15.40	11	54.99	0.88	0.02
4337	VBBM067	M	S	No	39.1	657.06	0.40	11	983.53	12.69	37.50
6411	VEKF017	F	S	No	25.7	700.12	14.80	5	972.00	2.42	4.38
6412	VDF171	F	S	No	9.3	537.13	14.80	2	869.78	13.51	9.09
4857	VBBM081	M	S	No	30.8	784.77	8.80	10	1808.47	2.44	10.91
5732	VLF184	F	S	No	40.4	681.14	0.00	3	307.23	0.28	0.73

5906	VJXF035	F	S	No	46.6	749.09	1.60	10	429.73	0.78	2.28
5760	VBBM076	M	D	No	36.6	708.86	0.20	3	923.36	2.63	15.31
6021	VFAM002	M	S	No	9.8	540.22	1.60	16	352.81	0.62	1.90
3713	VVHM011	M	S	No	68.2	646.92	6.40	21	na	1.91	30.25

Table S5. Raw faecal data

Sample number	Subject	Sex	Rank	Breeding	Age (mo)	AM vs. PM	Mean weight (g)	Total rainfall (mm/mo)	FAM (ng/g faeces)
44488	VFF157	F	D	no	46.9	PM	666.00	9.00	388.25
40456	VCDF002	F	D	no	83.9	AM	860.3846154	53.40003	378.100
40554	VTYF035	F	D	no	16.9	AM	614.62	43.40	329.50
41207	VDF115	F	D	no	66.6	AM	636.91	8.20	324.43
41154	VTYF035	F	D	no	16.8	AM	631.75	24.99999	299.000
40053	VWF133	F	D	no	38.2	AM	688.54	26.00	275.10
43569	VDF115	F	D	no	74.1	AM	771.1	11.199995	273.800
40992	VWF063	F	D	no	96.8	AM	783.875	20.399998	269.900
49190	VVHF035	F	D	no	39.9	AM	853.9411765	41.600022	256.000
40477	VCDF002	F	D	no	83.9	PM	860.3846154	53.40003	246.550
43678	VDF115	F	D	no	74.1	PM	767.7777778	11.199995	245.633
465523	VSQF012	F	D	no	42.1	PM	700.16	1.80	237.70
40530	VFF138	F	D	no	49.7	PM	891	24.99999	232.050
42943	VDF115	F	D	no	70.5	AM	678.45	27.20	229.9
40627	VFF138	F	D	no	50.1	PM	861.5	68.399985	227.900
47581	VLF134	F	D	no	72.2	AM	1000.285714	42.000022	206.500
466055	VSQF012	F	D	no	42.9	AM	774.07	78.20	205.70
45298	VLF111	F	D	no	94.8	AM	722.43	6.40	199.45
49670	VKUF065	F	D	no	31.3	AM	573.31	0.20	190.70
41652	VDF115	F	D	no	66.9	AM	613.80	12.60	188.53
466786	VRRF116	F	D	no	54.6	AM	798.00	0.00	184.18
41011	VDF115	F	D	no	66.4	AM	681.50	20.40	178.05
40564	VFF138	F	D	no	49.8	AM	887.4285714	51.799987	171.133
43668	VDF115	F	D	no	74.3	AM	748.25	12.199995	171.000
48778	VKUF005	F	D	no	75.3	AM	806.7142857	0.4	162.650
466197	VGGF014	F	D	no	113.0	AM	672.18	78.20	152.95
466199	VGGF014	F	D	no	113.0	PM	672.18	78.20	152.53
45364	VJXF008	F	D	no	53.7	AM	732.07	4.20	140.25
39840	VWF133	F	D	no	37.6	PM	689.67	4.20	134.40
48085	VKUF065	F	D	no	24.9	PM	626.40	38.00	126.05
39950	VWF133	F	D	no	38.0	PM	664.67	26.40	124.27
465840	VGGF014	F	D	no	112.7	AM	643.31	78.20	118.90
44372	VWF075	F	D	no	99.0	PM	803.9230769	9.000001	117.900
45224	VJXF008	F	D	no	53.5	PM	747.21	6.40	113.80
40522	VTYF035	F	D	no	16.8	PM	627.3846154	24.99999	110.300
47095	VDF115	F	D	no	83.4	AM	717.83	0.00	106.35
40357	VTYF035	F	D	no	16.5	AM	671.5	68.20003	104.100
40394	VTYF035	F	D	no	16.6	AM	661.3	90.40002	102.150
40354	VCDF002	F	D	no	83.6	AM	871.6923077	68.20003	100.400
40523	VTYF035	F	D	no	16.9	PM	624.14	25.00	99.50
40372	VTYF035	F	D	no	16.5	PM	671.5	68.20003	98.300
40440	VLF102	F	D	no	85.7	PM	954.1538462	90.40002	85.967
44507	VSQF005	F	D	no	48.7	AM	733.88	9.00	82.08
48935	VKUF042	F	D	no	43.2	PM	782.10	14.00	80.50
41279	VLF111	F	D	no	84.5	PM	675.75	6.20	78.00
41315	VDF115	F	D	no	66.7	PM	629.44	6.20	77.20
40994	VDF115	F	D	no	66.4	AM	681.50	20.40	73.27
52437	VWF177	F	D	no	33.9	PM	747.50	1.40	66.90
44737	VWF133	F	D	no	50.8	AM	680.2857143	5.8	64.650
40561	VTYF035	F	D	no	17.0	AM	600.38	51.80	63.08
44619	VWF133	F	D	no	50.5	PM	691	11.600001	60.925

51824	VUKF007	F	D	no	17.1	AM	706.60	4.40	60.85
40446	VLF102	F	D	no	85.7	AM	954.1538462	73.20003	56.567
60004	VUKF007	F	D	no	17.9	AM	664.28	1.80	56.50
44002	VWF093	F	D	no	87.2	AM	736.2307692	2.2	48.200
46435	VWF147	F	D	no	39.4	PM	539.92	4.00	40.45
44607	VWF133	F	D	no	50.5	AM	691	11.600001	39.514
40395	VTYF035	F	D	no	16.6	AM	661.3	90.40002	36.900
43612	VDF115	F	D	no	74.2	AM	767.7777778	11.199995	34.950
40471	VDF115	F	D	yes	64.8	AM	736.20	25.00	443.10
44584	VKUF005	F	D	yes	59.2	PM	682.24	10.20	376.90
47441	VBBF069	F	D	yes	34.4	AM	840.41	6.00	365.90
40692	VWF133	F	D	yes	40.1	PM	851.00	68.20	363.00
44919	VSQF012	F	D	yes	40.0	PM	791.18	6.80	358.55
39725	VCDF002	F	D	yes	81.8	PM	763.94	0.40	327.90
40317	VLF102	F	D	yes	85.4	PM	963.30	66.60	312.30
44775	VRRF116	F	D	yes	50.2	PM	799.93	8.80	289.60
39963	VDF115	F	D	yes	63.3	AM	598.64	26.40	254.98
43727	VSQF012	F	D	yes	37.8	PM	884.30	12.20	239.75
47803	VKUF065	F	D	yes	25.2	AM	637.07	40.80	236.38
39947	VWF093	F	D	yes	75.6	AM	648.53	26.40	229.20
43408	VDF115	F	D	yes	73.5	AM	782.89	11.80	227.68
43350	VDF115	F	D	yes	73.4	AM	766.44	11.80	227.25
40235	VTYF035	F	D	yes	16.1	AM	663.80	86.60	227.00
40826	VWF063	F	D	yes	96.3	AM	817.17	60.00	224.400
40253	VTYF035	F	D	yes	16.1	PM	672.33	86.60	224.00
45063	VLF102	F	D	yes	97.3	AM	780.27	7.80	223.38
44655	VLF102	F	D	yes	96.9	AM	776.27	11.60	221.58
40240	VTYF035	F	D	yes	16.1	PM	663.80	86.60	220.40
43559	VWF093	F	D	yes	86.3	PM	715.83	10.00	209.40
40270	VLF102	F	D	yes	85.2	PM	931.55	65.60	201.95
47418	VKUF039	F	D	yes	39.6	PM	807.14	5.80	183.05
40711	VWF133	F	D	yes	40.2	AM	862.67	70.00	175.90
43425	VDF115	F	D	yes	73.6	PM	791.75	10.00	167.50
43667	VWF093	F	D	yes	86.6	AM	797.64	12.20	164.40
47573	VKUF005	F	D	yes	71.3	AM	855.00	34.00	163.08
45365	VSQF012	F	D	yes	40.3	PM	817.18	6.40	155.28
48076	VVHF035	F	D	yes	41.4	AM	729.60	45.80	154.75
39829	VCDF002	F	D	yes	82.0	PM	707.57	0.40	150.20
43575	VSQF012	F	D	yes	37.5	AM	831.00	11.20	149.30
48279	VKUF065	F	D	yes	26.6	AM	711.00	97.00	146.80
40851	VDF115	F	D	yes	65.0	AM	770.71	51.80	142.90
43353	VDF115	F	D	yes	73.4	AM	766.44	11.80	142.80
48283	VKUF065	F	D	yes	26.6	PM	711.00	97.00	138.350
39759	VCDF002	F	D	yes	81.9	AM	741.00	0.40	138.20
47887	VKUF065	F	D	yes	25.5	AM	659.06	48.80	135.83
40200	VFF138	F	D	yes	48.9	PM	793.50	87.60	133.40
40331	VFF138	F	D	yes	49.3	PM	894.43	68.20	132.45
47386	VVHF035	F	D	yes	38.5	AM	701.60	6.00	131.35
44200	VWF147	F	D	yes	36.4	AM	721.33	9.00	129.17
43422	VDF115	F	D	yes	73.6	PM	791.75	10.00	127.23
47122	VWF177	F	D	yes	18.4	PM	585.35	0.00	119.35
47433	VRRF137	F	D	yes	34.5	AM	800.90	6.00	115.95
44617	VLF102	F	D	yes	96.7	PM	778.93	11.60	115.80
40314	VWF133	F	D	yes	39.0	AM	765.86	66.60	111.10
48261	VKUF065	F	D	yes	26.6	AM	711.50	97.00	109.33
45279	VSQF012	F	D	yes	40.4	AM	817.18	6.40	108.25

43768	VSQF012	F	D	yes	38.0	PM	894.94	2.20	107.43
40327	VFF138	F	D	yes	49.3	AM	894.43	68.20	103.17
44523	VLF102	F	D	yes	96.6	AM	779.50	9.00	90.10
39782	VCDF002	F	D	yes	81.9	PM	735.07	0.40	88.93
466780	VSQF012	F	D	yes	43.9	PM	815.22	0.00	88.43
47811	VKUF065	F	D	yes	25.2	PM	637.07	40.80	86.04
43497	VWF093	F	D	yes	86.2	AM	706.36	10.00	81.80
40681	VWF133	F	D	yes	40.1	AM	851.00	68.20	78.90
44629	VLF102	F	D	yes	97.0	PM	777.00	8.60	76.23
47109	VAZF029	F	D	yes	32.5	PM	791.47	0.00	68.90
43993	VSQF012	F	D	yes	38.3	PM	931.59	2.20	67.97
43599	VWF093	F	D	yes	86.4	PM	749.00	11.20	66.23
40445	VDF115	F	D	yes	64.7	PM	736.20	73.20	65.05
40713	VWF133	F	D	yes	40.2	AM	862.67	70.00	61.75
48064	VKUF065	F	D	yes	26.0	PM	682.95	61.80	59.70
47111	VLF102	F	D	yes	104.4	AM	818.81	0.00	51.23
40257	VTYF035	F	D	yes	16.1	AM	678.70	86.60	45.45
47393	VKUF005	F	D	yes	68.9	AM	811.17	0.00	43.24
47545	VKUF005	F	D	yes	71.1	PM	845.18	34.20	37.96
47147	VWF177	F	D	yes	18.6	AM	600.89	0.00	33.63
48340	VKUF065	F	D	yes	26.8	AM	733.07	79.20	28.03
49782	VVHM038	M	D	no	44.6	AM	653.44	0.20	182.750
47096	VDM129	M	D	no	55.9	AM	761.00	0.00	179.90
40258	VJXM010	M	D	no	38.3	AM	778.20	86.60	171.17
39826	VJXM005	M	D	no	39.6	AM	767.63	4.20	161.30
45087	VWM120	M	D	no	71.8	AM	638.88	9.00	151.95
40697	VWM120	M	D	no	60.7	PM	717.42	76.20	127.18
47395	VBBM044	M	D	no	49.9	AM	738.13	5.80	124.20
40279	VVHM001	M	D	no	48.4	AM	688.06	65.40	115.07
40663	VDM129	M	D	no	40.9	AM	771.38	67.20	114.10
44522	VJXM005	M	D	no	52.3	AM	782.86	9.00	108.70
40500	VJXM005	M	D	no	41.4	AM	807.85	90.40	108.30
48777	VRRM113	M	D	no	68.2	AM	791.00	0.40	106.30
47430	VEKM007	M	D	no	34.0	AM	747.00	6.00	103.25
48426	VLM155	M	D	no	62.5	AM	726.80	2.40	102.95
40373	VJXM010	M	D	no	38.6	PM	789.56	68.20	96.80
47661	VRRM113	M	D	no	64.8	AM	837.90	37.60	95.20
44551	VDM157	M	D	no	16.0	AM	577.00	11.60	95.10
45489	VCBM001	M	D	no	85.5	AM	704.33	2.60	93.00
47597	VEKM007	M	D	no	35.2	AM	808.46	41.60	90.850
465798	VJXM005	M	D	no	55.9	AM	709.00	78.20	90.75
43577	VWM132	M	D	no	48.8	AM	728.25	11.20	89.20
47094	VBBM070	M	D	no	31.8	PM	672.11	0.00	87.05
45388	VWM136	M	D	no	49.1	AM	714.73	4.20	85.23
43911	VWM132	M	D	no	49.2	PM	716.82	2.20	83.35
47867	VRRM113	M	D	no	65.2	AM	834.20	23.20	83.10
48836	VBBM044	M	D	no	55.9	AM	832.00	14.00	77.15
47558	VEKM007	M	D	no	35.0	PM	819.71	34.00	75.30
40468	VLM114	M	D	no	75.9	PM	831.00	53.40	70.40
40453	VJXM005	M	D	no	41.4	AM	807.85	73.20	67.77
40269	VDM125	M	D	no	46.2	PM	713.91	65.60	67.48
47656	VEKM007	M	D	no	35.5	PM	798.00	41.40	64.25
47480	VVHM038	M	D	no	36.3	AM	730.29	6.40	62.05
40633	VDM125	M	D	no	47.1	PM	756.80	57.40	59.45
40879	VDM129	M	D	no	41.6	AM	774.20	27.60	58.45
40667	VDM129	M	D	no	40.9	AM	771.38	67.20	58.05

40458	VCBM001	M	D	no	72.9	AM	811.50	53.40	58.03
47652	VEKM007	M	D	no	35.5	AM	798.00	41.40	56.55
40441	VJXM005	M	D	no	41.4	PM	807.85	90.40	56.50
43600	VVHM001	M	D	no	58.2	PM	698.64	11.20	55.70
40199	VDM125	M	D	no	46.0	PM	689.33	87.60	55.37
43613	VDM129	M	D	no	49.7	AM	731.38	11.20	50.80
40435	VJXM010	M	D	no	38.8	AM	794.90	73.20	48.90
40478	VCBM001	M	D	no	72.9	PM	811.50	53.40	48.83
41613	VDM129	M	D	no	49.7	AM	731.38	11.20	46.00
40362	VJXM005	M	D	no	41.2	PM	813.18	68.20	43.53
45110	VRRM123	M	D	no	46.2	PM	718.55	6.80	42.35
45748	VWM132	M	D	no	52.3	AM	606.67	2.20	42.15
45351	VDM129	M	D	no	52.6	AM	706.82	4.20	40.50
40857	VLM114	M	D	no	77.2	PM	820.83	33.20	38.25
47875	VVHM038	M	D	no	38.2	PM	725.88	38.00	36.35
45363	VWM136	M	D	no	49.0	PM	719.92	4.20	36.30
44468	VJXM005	M	D	no	52.2	AM	774.75	9.00	35.400
43728	VWM132	M	D	no	49.1	PM	722.67	12.20	33.05
40454	VJXM005	M	D	no	41.4	AM	807.85	73.20	32.33
40332	VVHM001	M	D	no	48.5	PM	697.86	68.20	31.00
45176	VRRM123	M	D	no	46.4	AM	718.38	6.40	28.40
47462	VVHM038	M	D	no	36.2	AM	723.85	6.40	27.95
44785	VWM143	M	D	no	37.5	PM	631.87	7.80	26.85
44578	VRRM113	M	D	no	51.9	AM	710.67	11.60	24.05
44652	VJXM005	M	D	yes	52.5	AM	798.60	11.60	229.900
40565	VDM125	M	D	yes	46.9	AM	738.55	51.80	180.03
45628	VSQM014	M	D	yes	40.8	PM	816.25	2.60	179.70
40606	VJXM005	M	D	yes	41.9	PM	810.73	57.40	173.35
44627	VJXM005	M	D	yes	52.7	PM	804.00	8.60	154.65
44569	VWM136	M	D	yes	47.7	AM	746.50	11.60	126.25
45522	VSQM014	M	D	yes	40.9	AM	810.80	2.60	108.20
40722	VJXM005	M	D	yes	42.3	AM	819.94	59.40	99.850
39865	VJXM005	M	D	yes	39.8	AM	767.44	5.20	97.65
41143	VJXM005	M	D	yes	41.9	AM	810.88	60.20	89.75
43335	VDM129	M	D	yes	48.9	AM	734.40	1.80	81.700
45892	VWM120	M	D	yes	73.0	AM	579.25	2.20	77.35
47103	VVHM031	M	D	yes	39.3	AM	650.00	0.00	63.25
45640	VDM129	M	D	yes	53.0	PM	696.83	2.60	58.70
45499	VSQM014	M	D	yes	40.8	PM	816.25	2.60	51.85
43763	VRRM123	M	D	yes	44.0	PM	834.36	12.20	39.90
44587	VLF152	F	S	no	46.8	AM	635.17	11.60	329.30
47485	VLF182	F	S	no	34.4	PM	672.00	6.00	272.70
47037	VJXF052	F	S	no	20.1	PM	581.44	0.00	269.20
43748	VAZF020	F	S	no	35.1	AM	721.00	2.20	261.30
46437	VWF161	F	S	no	25.7	PM	561.77	4.00	247.30
43385	VCDF002	F	S	no	92.6	PM	688.55	11.80	246.58
47044	VSQF035	F	S	no	25.6	AM	588.11	0.00	233.65
46898	VWF176	F	S	no	17.0	AM	539.43	0.00	232.10
47340	VLF184	F	S	no	30.4	AM	627.83	0.00	226.00
40254	VTYF031	F	S	no	22.3	PM	513.57	86.60	216.00
49394	VJXF035	F	S	no	42.8	AM	759.23	8.80	193.90
48181	VAZF052	F	S	no	24.1	PM	747.25	106.00	193.53
48907	VKUF055	F	S	no	37.3	AM	686.31	14.00	186.65
43428	VAZF015	F	S	no	37.2	PM	617.50	10.00	184.75
46725	VJXF052	F	S	no	18.1	PM	589.64	15.40	182.30
43296	VDF144	F	S	no	31.6	AM	639.09	1.80	181.98

45826	VSQF011	F	S	no	41.2	PM	754.07	2.20	170.03
465795	VWF177	F	S	no	12.8	PM	447.29	78.20	167.03
49194	VKUF055	F	S	no	36.1	AM	685.29	0.40	165.60
43596	VSQF011	F	S	no	37.6	PM	812.29	11.20	165.53
40157	VTYF030	F	S	no	22.0	PM	553.78	91.40	164.00
43578	VSQF011	F	S	no	37.5	AM	810.08	11.20	163.85
41021	VAZF028	F	S	no	15.9	AM	712.70	9.00	163.100
40241	VTYF030	F	S	no	22.3	PM	567.71	86.60	160.13
46739	VDF166	F	S	no	8.9	PM	466.43	15.40	157.25
45506	VTYF042	F	S	no	13.2	PM	424.47	2.60	155.38
50455	VVHF072	F	S	no	9.7	AM	587.24	0.20	155.00
53200	VVHF072	F	S	no	13.2	PM	664.8125	19.2	154.100
46897	VWF176	F	S	no	16.9	PM	541.64	0.00	151.45
43607	VSQF011	F	S	no	37.6	PM	821.93	11.20	150.13
40771	VAZF028	F	S	no	15.0	AM	726.17	60.00	149.80
466035	VJXF053	F	S	no	15.0	PM	502.25	78.20	143.45
40158	VWF161	F	S	no	11.4	AM	535.15	91.40	142.15
466840	VKUF065	F	S	no	16.1	PM	497.35	0.00	142.05
46881	VJXF053	F	S	no	19.0	AM	547.29	0.00	131.10
43673	VSQF035	F	S	no	15.5	PM	568.17	12.20	128.30
43189	VAZF029	F	S	no	21.8	AM	739.82	1.80	125.450
44542	VDF144	F	S	no	33.9	PM	612.83	9.00	124.95
43464	VAZF015	F	S	no	37.2	AM	617.50	10.00	122.05
53470	VVHF072	F	S	no	13.6	PM	589.43	22.20	121.75
43517	VSQF011	F	S	no	37.4	PM	800.92	10.00	120.10
40827	VAZF029	F	S	no	15.1	AM	710.93	60.00	113.83
41295	VAZF027	F	S	no	24.6	AM	594.4444444	6.2	113.250
465775	VJXF035	F	S	no	28.8	PM	646.33	78.20	112.05
52403	VBBF093	F	S	no	12.8	AM	625.58	2.00	111.45
46661	VWF176	F	S	no	15.9	PM	542.65	15.40	110.95
46973	VJXF052	F	S	no	19.0	PM	579.69	0.00	107.90
43200	VAZF020	F	S	no	33.3	AM	609.89	1.80	106.63
45631	VAZF052	F	S	no	10.9	PM	513.87	2.60	106.43
44586	VNEF037	F	S	no	10.1	PM	432.00	9.00	105.50
47093	VLF180	F	S	no	32.1	PM	596.08	0.00	101.23
43658	VKUF042	F	S	no	24.5	PM	682.82	11.20	100.95
46700	VBBF083	F	S	no	17.1	PM	527.80	15.40	99.35
43598	VAZF020	F	S	no	34.7	PM	682.22	11.20	95.83
43155	VSQF011	F	S	no	35.8	PM	772.78	0.00	92.900
46448	VAZF052	F	S	no	12.1	PM	507.12	3.60	92.53
45402	VRRF151	F	S	no	12.6	PM	690.5333333	4.2	92.375
43297	VDF144	F	S	no	31.6	AM	639.09	1.80	92.33
43247	VEKF008	F	S	no	20.0	AM	591.25	1.80	91.93
48220	VJXF057	F	S	no	17.4	AM	668.00	102.00	91.90
43969	VCDF002	F	S	no	93.9	PM	734.60	2.20	91.50
47323	VRRF153	F	S	no	21.8	AM	710.50	0.00	88.30
43913	VRRF153	F	S	no	9.9	PM	517.62	2.20	88.10
40833	VAZF028	F	S	no	15.1	PM	732.69	60.00	86.45
45972	VWF176	F	S	no	11.6	PM	454.93	2.80	80.35
45805	VRRF146	F	S	no	17.6	AM	541.63	2.20	80.13
46928	VJXF053	F	S	no	19.2	AM	552.50	0.00	78.25
465794	VWF176	F	S	no	12.8	PM	452.06	78.20	78.05
47101	VRRF151	F	S	no	19.3	AM	662.17	0.00	77.10
45502	VWF176	F	S	no	11.1	AM	464.69	2.60	75.93
43807	VAZF020	F	S	no	35.0	AM	693.17	12.20	75.90
43196	VAZF033	F	S	no	18.8	AM	591.70	1.80	75.78

45760	VRRF146	F	S	no	17.5	PM	574.78	3.40	70.90
50380	VVHF072	F	S	no	9.6	AM	588.67	1.60	68.65
44380	VBBF069	F	S	no	22.5	AM	611.70	9.00	67.68
46862	VWF176	F	S	no	17.0	AM	541.64	0.00	67.10
43777	VEKF010	F	S	no	21.6	AM	600.13	2.20	66.63
50479	VVHF070	F	S	no	9.7	AM	588.06	0.20	64.90
465612	VTYF042	F	S	no	14.4	AM	444.36	3.60	62.25
466981	VLF164	F	S	no	41.6	PM	626.40	0.00	61.65
467291	VBBF083	F	S	no	15.8	AM	502.60	0.00	60.93
43404	VEKF008	F	S	no	20.5	PM	589.92	10.00	60.68
46614	VJXF057	F	S	no	8.9	AM	391.50	15.40	60.33
43747	VKUF042	F	S	no	24.7	AM	672.47	2.20	58.40
40485	VTYF030	F	S	no	21.1	AM	493.38	4.20	56.58
44588	VEKF008	F	S	no	22.7	AM	572.10	11.60	55.83
45460	VRRF146	F	S	no	17.0	PM	648.1333333	4.2	55.825
45833	VRRF151	F	S	no	13.0	PM	594.40	2.20	55.50
43471	VSQF035	F	S	no	14.9	AM	526.00	10.00	54.40
48011	VWF176	F	S	no	24.0	AM	725.25	54.00	53.43
49108	VWF176	F	S	no	23.4	AM	823.1538462	48.79999	52.433
43646	VKUF042	F	S	no	24.4	AM	675.83	11.20	51.40
465802	VKUF065	F	S	no	14.9	PM	455.30	78.20	50.73
45750	VSQF035	F	S	no	18.7	AM	531.92	2.20	50.35
45555	VKUF065	F	S	no	12.8	PM	464.50	4.20	50.15
49354	VJXF057	F	S	no	20.8	AM	618	0.2	50.000
467542	VWF177	F	S	no	15.4	PM	599.00	0.00	49.75
43444	VSQF041	F	S	no	8.9	AM	419.83	10.00	49.20
45346	VRRF146	F	S	no	16.9	PM	667.2857143	6.4	45.925
466963	VAZF052	F	S	no	14.2	AM	619.42	0.00	44.78
47792	VBBF083	F	S	no	24.3	AM	613.57	40.80	43.733
43909	VKUF019	F	S	no	48.4	AM	535.29	2.20	43.17
43298	VDF144	F	S	no	31.5	PM	640.00	1.80	42.70
43706	VKUF065	F	S	no	10.0	AM	455.15	12.20	41.13
46067	VBBF083	F	S	no	12.2	PM	417.33	2.60	40.90
45487	VSQF011	F	S	no	40.8	AM	789.55	2.60	40.13
47112	VDF146	F	S	no	41.1	AM	710.43	15.40	39.18
49134	VBBF083	F	S	no	27.6	PM	737.6666667	0.2	38.150
46613	VJXF058	F	S	no	8.9	PM	376.13	15.40	37.90
45763	VAZF052	F	S	no	11.2	PM	510.36	2.20	37.85
45761	VRRF151	F	S	no	12.9	PM	632.94	3.40	36.35
50436	VBBF093	F	S	no	10.3	AM	648.00	1.40	36.13
43610	VDF144	F	S	no	32.5	AM	662.60	11.20	35.13
43402	VFF186	F	S	no	8.9	PM	321.70	10.00	34.88
48327	VWF176	F	S	no	24.8	AM	657.76	95.80	34.85
46299	VKUF065	F	S	no	13.9	AM	455.20	4.00	34.750
43348	VDF155	F	S	no	18.7	AM	578.79	11.80	33.83
43615	VEKF008	F	S	no	21.1	AM	620.36	11.20	32.23
45888	VTYF042	F	S	no	13.5	AM	424.79	2.20	30.55
467342	VWF176	F	S	no	14.9	PM	539.90	0.00	30.10
43160	VEKF010	F	S	no	19.4	PM	577.00	0.00	30.05
43787	VKUF042	F	S	no	24.7	PM	675.07	2.20	29.98
43143	VEKF008	F	S	no	19.2	PM	580.00	0.00	29.95
43438	VEKF008	F	S	no	20.6	AM	597.69	10.00	29.45
53384	VEKF022	F	S	no	15.4	PM	529.40	17.00	28.05
40733	VAZF029	F	S	no	14.8	AM	700.06	60.00	26.63
45861	VWF176	F	S	no	11.5	AM	458.85	2.20	22.20
43623	VDF144	F	S	no	32.5	PM	662.60	11.20	20.93

43371	VFF186	F	S	no	8.8	AM	320.91	11.80	20.70
45904	VTYF042	F	S	no	13.7	PM	428.69	2.80	20.08
47884	VWF176	F	S	no	23.5	AM	823.1538462	48.79999	18.350
46656	VKUF065	F	S	no	16.1	AM	494.47	0.00	17.68
43666	VVHF034	F	S	no	30.5	AM	761.71	12.20	15.92
49527	VEKF022	F	S	no	8.8	PM	478.82	0.00	15.68
43804	VEKF008	F	S	no	20.9	AM	603.40	10.00	14.03
47542	VRRF159	F	S	no	13.5	AM	643.06	5.40	13.68
45700	VKUF065	F	S	no	13.1	AM	461.08	2.60	12.83
40260	VWF161	F	S	yes	11.7	AM	568.36	86.60	340.60
45418	VAZF028	F	S	yes	26.4	AM	889.14	2.60	316.300
40991	VAZF030	F	S	yes	15.6	AM	791.11	20.40	264.900
52223	VVHF072	F	S	yes	11.9	AM	617.33	5.40	253.40
53266	VBBF088	F	S	yes	16.1	AM	644.36	18.20	227.55
52977	VVHF072	F	S	yes	12.1	AM	629.00	2.40	224.35
43669	VSQF011	F	S	yes	37.8	AM	834.00	12.20	221.00
39831	VSQF005	F	S	yes	36.0	AM	673.64	4.20	208.90
45722	VAZF028	F	S	yes	26.8	AM	891.38	2.20	197.38
45123	VAZF036	F	S	yes	22.7	PM	808.62	9.00	191.55
52844	VVHF072	F	S	yes	12.6	AM	680.87	4.60	183.35
47617	VKUF065	F	S	yes	23.7	AM	788.56	37.00	176.10
44557	VAZF030	F	S	yes	24.8	AM	755.31	11.60	175.75
40274	VKUF030	F	S	yes	25.2	AM	794.47	65.40	166.00
44763	VRRF146	F	S	yes	16.0	PM	612.79	8.80	164.20
53680	VBBF088	F	S	yes	17.2	AM	716.36	11.40	160.40
49905	VBBF083	F	S	yes	31.2	AM	656.80	0.00	144.30
44827	VRRF146	F	S	yes	16.4	AM	665.33	6.80	139.65
40715	VWF147	F	S	yes	26.8	PM	676.00	70.00	139.30
53371	VBBF093	F	S	yes	14.0	AM	660.77	17.40	126.75
45077	VRRF146	F	S	yes	16.6	PM	682.94	9.00	121.00
41124	VAZF030	F	S	yes	15.7	PM	822.00	9.40	117.87
47550	VRRF156	F	S	yes	12.9	AM	669.07	34.20	97.68
48856	VJXF057	F	S	yes	17.2	AM	634.00	14.00	87.03
47587	VRRF156	F	S	yes	13.2	AM	689.54	41.80	86.90
47746	VWF176	F	S	yes	21.9	AM	748.57	41.60	82.850
44926	VRRF151	F	S	yes	11.9	PM	679.50	6.80	75.65
40081	VKUF030	F	S	yes	24.5	PM	654.64	26.00	74.38
39781	VSQF005	F	S	yes	35.9	PM	682.36	0.40	69.28
53443	VBBF093	F	S	yes	14.2	AM	669.58	22.20	61.10
45151	VRRF151	F	S	yes	12.1	AM	715.18	6.40	59.43
49011	VJXF057	F	S	yes	17.7	PM	663.00	8.80	59.40
50232	VLF206	F	S	yes	13.7	PM	548.93	1.60	57.40
40237	VWF161	F	S	yes	11.6	PM	551.62	86.60	55.30
44560	VRRF151	F	S	yes	11.0	AM	562.81	11.60	42.10
39832	VSQF005	F	S	yes	36.0	AM	673.64	4.20	41.47
50450	VLF206	F	S	yes	14.1	AM	582.08	1.40	40.60
46844	VBBF083	F	S	yes	17.0	PM	537.11	0.00	34.70
47557	VRRF156	F	S	yes	13.0	PM	674.36	34.00	27.85
49200	VJXF057	F	S	yes	17.5	PM	634.00	14.20	22.60
47039	VBBF083	F	S	yes	18.3	AM	622.67	0.00	16.38
44512	VJXM054	M	S	no	11.1	PM	493.09	9.00	193.58
44608	VSQM039	M	S	no	11.0	PM	458.54	11.60	176.13
44538	VLM157	M	S	no	39.2	AM	649.47	9.00	176.00
44537	VTYM045	M	S	no	11.4	PM	459.05	9.00	149.58
44543	VDM145	M	S	no	33.8	AM	666.00	9.00	148.40
44487	VDM145	M	S	no	33.9	AM	666.00	9.00	146.10

45280	VSQM040	M	S	no	12.1	AM	470.55	6.40	143.43
44558	VAZM050	M	S	no	9.2	AM	420.22	11.60	139.53
49513	VBBM090	M	S	no	10.5	AM	645.00	0.20	132.83
45216	VWM175	M	S	no	10.4	PM	419.38	6.40	125.10
46502	VAZM051	M	S	no	12.1	PM	499.06	3.60	123.80
49043	VBBM074	M	S	no	33.4	AM	718.38	8.80	121.90
45297	VSQM040	M	S	no	12.1	PM	470.55	6.40	121.28
44503	VAZM051	M	S	no	9.1	PM	484.40	9.00	117.60
45281	VSQM039	M	S	no	12.2	AM	471.55	6.40	115.23
46702	VBBM084	M	S	no	17.1	PM	520.67	15.40	114.28
47099	VVHM052	M	S	no	21.7	AM	540.00	0.00	113.10
44562	VBBM025	M	S	no	37.7	AM	682.33	12.00	111.05
45227	VWM175	M	S	no	10.4	PM	421.00	6.40	108.43
467262	VVHM060	M	S	no	16.3	AM	481.11	0.00	108.10
44466	VJXM054	M	S	no	11.1	AM	490.60	9.00	106.25
466033	VJXM055	M	S	no	15.0	PM	503.11	78.20	105.68
45719	VSQM037	M	S	no	13.4	AM	491.07	2.20	101.38
47747	VBBM085	M	S	no	24.0	AM	584.36	38.00	100.050
467282	VSQM039	M	S	no	16.3	PM	565.17	0.00	97.65
45075	VVHM060	M	S	no	11.8	AM	426.25	6.80	97.63
47879	VBBM074	M	S	no	28.7	PM	662.75	8.00	95.60
45393	VSQM037	M	S	no	12.9	PM	517.86	4.20	92.13
46398	VSQM037	M	S	no	14.1	PM	484.83	3.60	87.53
46393	VSQM039	M	S	no	13.6	AM	466.32	3.60	86.85
48783	VLM189	M	S	no	31.7	AM	659.50	2.00	86.15
44447	VWM175	M	S	no	9.1	AM	404.79	9.00	82.40
45288	VVHM060	M	S	no	12.1	AM	432.20	6.40	81.88
44520	VJXM037	M	S	no	22.5	AM	594.60	9.00	80.35
467481	VAZM051	M	S	no	15.1	AM	571.26	0.00	80.20
44625	VSQM040	M	S	no	11.2	PM	458.62	8.60	79.13
43531	VJXM055	M	S	no	9.3	AM	463.50	10.00	78.45
47864	VBBM074	M	S	no	29.5	AM	668.56	44.80	74.85
53468	VVHM071	M	S	no	13.6	PM	553.07	22.20	74.40
466705	VSQM027	M	S	no	32.7	PM	709.36	0.00	73.200
467060	VVHM056	M	S	no	15.9	PM	537.75	0.00	70.50
466571	VSQM037	M	S	no	16.0	AM	578.93	0.00	69.85
45286	VVHM056	M	S	no	12.1	AM	487.40	6.40	67.98
50446	VLM147	M	S	no	71.4	PM	681.87	1.40	66.600
45246	VJXM054	M	S	no	12.3	AM	542.50	6.40	66.48
467537	VJXM055	M	S	no	17.3	AM	549.15	0.00	66.45
45603	VWM155	M	S	no	30.2	PM	603.15	2.60	65.250
47102	VVHM038	M	S	no	32.0	AM	649.75	0.00	64.60
467298	VSQM042	M	S	no	16.3	PM	582.30	0.00	64.30
43487	VFM184	M	S	no	9.1	AM	367.33	10.00	61.38
48388	VWM131	M	S	no	66.0	AM	740.00	49.00	59.90
43401	VFM187	M	S	no	8.9	PM	330.90	10.00	59.48
46676	VJXM055	M	S	no	16.9	AM	543.33	0.00	58.05
47392	VBBM081	M	S	no	24.2	AM	701.38	5.80	57.85
46977	VAZM051	M	S	no	17.0	AM	559.07	0.00	57.15
465776	VJXM054	M	S	no	14.8	PM	521.00	78.20	56.55
43294	VJXM054	M	S	no	8.8	AM	431.00	1.80	56.38
45503	VWM175	M	S	no	11.1	AM	425.00	2.60	53.23
46471	VSQM040	M	S	no	13.7	PM	458.89	3.60	50.80
467252	VWM175	M	S	no	14.7	AM	490.06	0.00	46.70
43459	VSQM039	M	S	no	9.0	AM	408.72	10.00	46.25
43372	VFM184	M	S	no	8.8	AM	354.09	11.80	44.68

45296	VVHM056	M	S	no	12.2	AM	485.91	6.40	44.28
45488	VWM175	M	S	no	11.0	PM	426.40	2.60	42.68
50931	VKUM070	M	S	no	10.3	AM	572.56	14.80	42.38
45891	VEKM012	M	S	no	12.3	PM	542.70	2.20	39.05
45436	VSQM039	M	S	no	12.2	PM	471.55	6.40	38.30
46252	VLM189	M	S	no	18.0	PM	481.60	2.80	35.350
47754	VBBM085	M	S	no	24.0	PM	584.36	38.00	34.87
47490	VPAM010	M	S	no	13.2	AM	533.36	6.40	34.700
44556	VSQM013	M	S	no	39.1	AM	789.38	11.60	31.400
53367	VBBM090	M	S	no	16.6	AM	721.25	17.40	31.23
47880	VBBM085	M	S	no	24.6	PM	603.08	48.80	30.72
48072	VEKM012	M	S	no	24.7	PM	714.00	32.60	30.20
44510	VVHM056	M	S	no	10.8	AM	441.59	9.00	27.03
43608	VSQM039	M	S	no	9.4	AM	444.17	11.20	25.53
48771	VBBM066	M	S	no	39.8	PM	771.55	14.00	25.30
45876	VSQM039	M	S	no	12.9	PM	465.47	2.20	25.03
45862	VSQM040	M	S	no	12.9	AM	449.00	2.20	22.33
44955	VTYM045	M	S	no	11.8	AM	459.73	8.80	22.15
43603	VTYM045	M	S	no	10.0	AM	449.88	11.20	21.58
46348	VTYM045	M	S	no	11.0	PM	461.21	9.00	21.50
45714	VSQM037	M	S	no	13.3	PM	494.13	3.40	20.10
43902	VVHM060	M	S	no	9.9	AM	416.75	2.20	18.68
43494	VSQM042	M	S	no	9.1	AM	474.47	10.00	18.00
50314	VLM210	M	S	no	9.2	AM	545.67	1.60	15.05
47090	VBBM068	M	S	yes	31.8	AM	660.80	0.00	223.10
47091	VWM171	M	S	yes	24.6	PM	501.94	0.00	214.800
48969	VBBM084	M	S	yes	27.9	AM	688.73	8.80	171.70
47092	VAZM044	M	S	yes	22.5	PM	619.17	0.00	171.450
46933	VSQM042	M	S	yes	18.8	AM	624.21	0.00	171.40
45731	VJXM054	M	S	yes	13.2	AM	535.00	2.20	163.60
47104	VBBM006	M	S	yes	66.0	AM	731.70	15.40	149.60
47108	VKUM059	M	S	yes	24.0	AM	621.25	0.00	128.25
40120	VLM114	M	S	yes	74.8	AM	749.78	26.00	126.90
46139	VWM175	M	S	yes	11.8	PM	417.77	4.00	117.68
45756	VAZM050	M	S	yes	11.1	PM	477.33	2.20	109.58
46042	VVHM060	M	S	yes	13.8	AM	416.86	1.80	107.30
46507	VVHM056	M	S	yes	13.8	AM	466.38	1.80	106.78
53433	VBBM089	M	S	yes	16.8	AM	712.80	22.20	105.35
44511	VVHM060	M	S	yes	10.8	AM	396.11	9.00	100.28
45811	VBBM084	M	S	yes	12.3	AM	407.00	2.20	71.10
46427	VWM175	M	S	yes	12.1	AM	414.54	3.60	64.80
47087	VAZM051	M	S	yes	18.0	PM	567.92	0.00	61.90
45341	VAZM051	M	S	yes	10.7	AM	530.64	4.20	61.33
466135	VTYM045	M	S	yes	15.5	PM	469.86	78.20	60.90
465828	VSQM037	M	S	yes	14.8	PM	494.54	79.40	60.18
467461	VSQM039	M	S	yes	16.7	AM	565.00	0.00	58.05
48385	VLM181	M	S	yes	38.7	AM	705.57	56.80	56.50
45430	VWM175	M	S	yes	11.7	AM	420.17	3.80	54.85
46641	VVHM060	M	S	yes	17.2	AM	476.46	0.00	53.95
47440	VBBM071	M	S	yes	34.4	AM	704.07	6.00	45.55
467346	VVHM060	M	S	yes	15.8	PM	483.14	0.00	42.50
46682	VWM175	M	S	yes	15.9	PM	497.44	15.40	32.80
45912	VWM175	M	S	yes	11.5	AM	422.64	2.80	32.30
47408	VBBM074	M	S	yes	26.6	PM	665.17	5.00	31.20
46648	VSQM037	M	S	yes	18.0	PM	604.17	15.40	30.93
50326	VBBM092	M	S	yes	10.0	PM	577.17	1.60	25.00
